# A transcriptomic analysis of bermudagrass (*Cynodon dactylon*) provides novel insights into the basis of low temperature tolerance

**DOI:** 10.1186/s12870-015-0598-y

**Published:** 2015-09-11

**Authors:** Liang Chen, Jibiao Fan, Longxing Hu, Zhengrong Hu, Yan Xie, Yingzi Zhang, Yanhong Lou, Eviatar Nevo, Jinmin Fu

**Affiliations:** Key Laboratory of Plant Germplasm Enhancement and Specialty Agriculture and Wuhan Botanical Garden, Chinese Academy of Sciences, Wuhan, Hubei 430074 China; University of Chinese Academy of Sciences, 19 Yuquan Road, Beijing, 100049 China; College of Horticulture and Forestry Science, Huazhong Agricultural University, Wuhan, Hubei 430070 China; Institute of Evolution, University of Haifa, Mount Carmel, Haifa 31905 Israel

## Abstract

**Background:**

Cold stress is regarded as a key factor limiting widespread use for bermudagrass (*Cynodon dactylon*). Therefore, to improve cold tolerance for bermudagrass, it is urgent to understand molecular mechanisms of bermudagrass response to cold stress. However, our knowledge about the molecular responses of this species to cold stress is largely unknown. The objective of this study was to characterize the transcriptomic response to low temperature in bermudagrass by using RNA-Seq platform.

**Results:**

Ten cDNA libraries were generated from RNA samples of leaves from five different treatments in the cold-resistant (R) and the cold-sensitive (S) genotypes, including 4 °C cold acclimation (CA) for 24 h and 48 h, freezing (−5 °C) treatments for 4 h with or without prior CA, and controls. When subjected to cold acclimation, global gene expressions were initiated more quickly in the R genotype than those in the S genotype. The R genotype activated gene expression more effectively in response to freezing temperature after 48 h CA than the S genotype. The differentially expressed genes were identified as low temperature sensing and signaling-related genes, functional proteins and transcription factors, many of which were specifically or predominantly expressed in the R genotype under cold treatments, implying that these genes play important roles in the enhanced cold hardiness of bermudagrass. KEGG pathway enrichment analysis for DEGs revealed that photosynthesis, nitrogen metabolism and carbon fixation pathways play key roles in bermudagrass response to cold stress.

**Conclusions:**

The results of this study may contribute to our understanding the molecular mechanism underlying the responses of bermudagrass to cold stress, and also provide important clues for further study and in-depth characterization of cold-resistance breeding candidate genes in bermudagrass.

**Electronic supplementary material:**

The online version of this article (doi:10.1186/s12870-015-0598-y) contains supplementary material, which is available to authorized users.

## Background

Low temperature is one of the major limiting factors for the distribution, growth, and development of many plant species [1]. Breeding for increased cold hardiness in plants is an effective method to reduce the loss caused by cold stress. However, the lack of knowledge on the molecular mechanism of cold response in most plant species limits breeding progress. Therefore, elucidating the molecular mechanisms of plant responses to cold stress will accelerate the pace of genetic improvement of freezing tolerance.

When exposed to non-freezing temperatures for a certain period of time, plants show increased freezing tolerance by an adaptive phenomenon known as cold acclimation, which involves a number of biochemical and physiological changes [2, 3]. These intracellular changes are associated with alteration in gene expression. Currently, the well known cold signaling pathway is the ICE1-CBF-COR transcriptional cascade. In this pathway, C-repeat (CRT)-binding factors (CBFs) are rapidly induced by cold, and recognize the promoter regions of *COR* genes to activate their transcription [3, 4]. The expression of *CBF* is activated by ICE1 (inducer of *CBF* expression 1), which encodes a MYC-type bHLH transcription factor [4]. Transcriptome analysis also showed that only 12 % of the cold responsive genes are controlled by CBFs [5], suggesting that there were CBF-independent components involved in cold signaling. For example, loss function of *HOS9* gene encoding a homeobox transcription factor causes reduced freezing tolerance without changing the expression of *CBFs* and their target genes [6]. Although much progress has been made toward elucidating the molecular mechanisms of plant responses to cold stress, how plants sense low temperature signals remain unanswered. The recent findings support the hypothesis that plant cells can perceive cold stress and subsequently trigger the production of second messengers, such as Ca^2+^ via membrane rigidification [7].

In recent years, the RNA-Seq has become a key technology for investigating transcriptome profiling among different species by *de novo* assembly or mapping. Besides, RNA-Seq is an efficient means to generate functional genomic data for non-model organisms or those with genome characteristics extremely difficult to whole-genome sequencing [8, 9]. For instance, RNA-Seq has been successfully applied to characterize the transcriptomic response to low temperature in Chrysanthemum (*Chrysanthemum morifolium*), lily (*Lilium lancifolium*) and tea (*Camellia sinensis*) [10–12].

Bermudagrass [*Cynodon dactylon* (L). Pers.] is one of the most widely used warm-season turfgrass species for parks, lawns, and sport fields especially in golf courses [13, 14]. Bermudagrass displays high tolerance to salt, drought and heat stresses, but is sensitive to cold stress [15, 16]. Cold stress is a key factor limiting widespread use of bermudagrass. Thus, it is important to improve cold tolerance for bermudagrass. Although previous studies have identified several physiological and metabolic changes in bermudagrass after cold treatment, including the expression of genes encoding chitinase, dehydrin and antioxidant enzyme, protein synthesis, amino acid metabolism [15–20], the physiological and molecular mechanism of cold stress response in bermudagrass is largely unknown.

To date, few studies have been carried out to the transcriptional studies in bermudagrass. The transcriptomic responses of bermudagrass to low temperature using RNA-Seq have not been reported so far. In this study, the RNA-Seq platform based on Illumina NGS technology was used to investigate the transcriptomic response to low temperature by comparing the different transcriptome between two cold contrasting bermudagrass genotypes (Cold-resistant and -sensitive) subjected to periods of sub-zero temperature with or without a prior CA. Thus, the objectives of the present study were to (a) identify genes involved in response to chilling/freezing; (b) elucidate the molecular mechanisms of cold tolerance through transcriptomic analysis of the two genotypes differing in tolerance to cold stress; (c) gain a deep insight into the molecular basis of CA process in enhancing plant freezing tolerance.

## Methods

### Plant materials and growth conditions

The 128 bermudagrass accessions were planted in the plastic pots (15 cm diameter and 20 cm tall) filled with matrix (brown coal soil: sand 1:1). Each accession was repeated 3 times. The plants were treated with 4 °C for 21 d, and the plants cultivated under 30/25 °C (day/night) were set as the control. Transpiration rate and growth rate of the plants were determined every week. The membership function method of fuzzy mathematics was analyzed using the phenotypic traits after a 21 d chilling treatment. The membership values of each accession were the index of cold tolerance. After the first round screening, 5 relatively cold-tolerant and 5 cold-sensitive accessions were obtained, respectively. To further screen the relatively most cold-tolerant and cold-sensitive genotypes, the 10 accessions were treated with −5 °C for 4 h with or without cold acclimation. Finally, the most promising cold-tolerant (R) and -sensitive(S) bermudagrass genotypes were selected and further confirmed, respectively (Additional file 1).

The cold-tolerant (R) and -sensitive(S) bermudagrass plants were grown in plastic pots with a mix of sand and peat soil (1/1, v/v) in the greenhouse with natural sunlight, relative humidity of 87 %, and temperatures of 30/20 °C (day/night). The plants in pots are ramets of the same clone, and the genetic background for these plants is uniform. After two months of establishment, plants were transferred to controlled-environment growth chambers (HP300GS-C; Ruihua Instrument, Wuhan, China), with a 14-h photoperiod, photosynthetically active radiation at 450 μmol m^−2^ s^−1^ in the canopy level with a day/night temperature of 30/20 °C and 70 % humidity. Plants were fertilized three times a week with half-strength Hoagland’s solution until dripping throughout the experiment in order to keep them close to field capacity.

### Treatments and experimental design

When allowed to acclimate for 3 days at normal condition, plants were exposed to various cold treatments. The cold-tolerant and -sensitive genotypes were divided into two groups (Group I, II). Plants in Group I were placed in a freezing chamber set to 4 °C for 48 h before being transferred to −5 °C for 4 h, whereas plants in Group II without CA were directly incubated at −5 °C for 4 h. The leaf samples for transcriptome sequencing were collected at 0 h (named CdR_0, CdS_0), 24 h (CdRCA_24, CdSCA_24) and 48 h (CdRCA_48, CdSCA_48) after 4 °C treatment, −5 °C for 4 h after prior CA (CdRCA_4, CdSCA_4), and −5 °C for 4 h without prior CA (CdRNA_4, CdSNA_4), respectively. At each sampling time point, the leaves from three pots (three replicates) of each genotype were pooled together as one biological replicate and frozen immediately with liquid nitrogen, and stored at −80 °C in preparation for RNA-Seq analysis. There were ten samples in total used for Illumina Genome Analyzer deep sequencing.

### RNA preparation

Total RNA was isolated from the leaves using TRIzol reagent according to the manufacturer’s protocol (Invitrogen, CA, USA). Then, RNA degradation and contamination was monitored on 1 % agarose gels. RNA purity was checked using the Nano Photometer spectrophotometer (IMPLEN, CA, USA). The RNA concentration was measured using Qubit RNA Assay Kit in Qubit 2.0 Flurometer (Life Technologies, CA, USA). RNA integrity was evaluated using the RNA Nano 6000 Assay Kit of the Bioanalyzer 2100 system (Agilent Technologies, Santa Clara, CA, USA).

### Transcriptome sample preparation and sequencing

The total amount of 3 μg RNA per sample confirmed for RIN values above 8.0 was used as input material in constructing the sequencing library. The library was generated using Illumina TruSeq RNA Sample Preparation Kit (Illumina, San Diego, CA, USA) according to manufacturer’s recommendations, and ten index codes were added to the sample for subsequent documentation. Briefly, mRNA was purified from total RNA using poly-T oligo-attached magnetic beads. Fragmentation was carried out using divalent cations under elevated temperature in Illumina proprietary fragmentation buffer. First-strand cDNA was synthesized using random oligonucleotides and SuperScript II. Second-strand cDNA synthesis was subsequently performed using DNA polymerase I and RNase H. Remaining overhangs were converted into blunt ends via exonuclease/polymerase activities and enzymes were removed. After adenylation of 3’ ends of DNA fragments, Illumina PE adapter oligonucleotides were ligated to prepare for hybridization. To select cDNA fragments of preferentially 200 bp in length, the library fragments were purified with AMPure XP system (Beckman Coulter, Beverly, MA, USA). DNA fragments with ligated adaptor molecules on both ends were selectively enriched using Illumina PCR Primer Cocktail in a 10 cycle PCR. Products were purified (AMPure XP system) and quantified using the Agilent high-sensitivity DNA assay on the Agilent Bioanalyzer 2100 system. The clustering of the index-coded sample was performed on a cBot Cluster Generation System using TruSeq PE Cluster Kit v3-cBot-HS (Illumia) according to the vender’s instructions. After cluster generation, the library preparation was sequenced on an Illumina Hiseq 2000 platform and 100 bp single-end reads were generated.

### Bioinformatic analysis

#### Quality control

The raw reads were processed by removing reads containing adapter, reads containing ploy-N and low quality reads, and then the clean data (clean reads) were obtained. At the same time, Q20, Q30, GC-content and sequence duplication level of the clean data were calculated. All the downstream analyses were based on clean data with high quality.

#### Transcriptome assembly

The left files (read1 files) from all libraries/samples were pooled into one big left.fq file, and right files (read2 files) into one big right.fq file. Transcriptome assembly was accomplished based on the left.fq and right.fq using Trinity [21] with min_kmer_cov set to 2 by default and all other parameters set default.

#### Gene functional annotation

Gene function was annotated using the nucleotide (Nt) and protein (Nr, Pfam and Swiss-Prot) database, and assigned to functional categories in the KOG/COG, GO and KEGG database by searching BLASTx with an E value cutoff of 10^−5^.

#### Differential expression analysis

Prior to differential gene expression analysis, for each sequenced library, the read counts were adjusted by edgeR program package through one scaling normalized factor. Differential expression analysis of two samples was performed using the DEGseq (2010) R package. *P*-value was adjusted using q value [22]. *q* value < 0.005 & |log2(foldchange)| > 1 was set as the threshold for significant differential expression.

#### GO enrichment analysis

Gene Ontology (GO) enrichment analysis of the differentially expressed genes (DEGs) was implemented by the GOseq R packages based on Wallenius non-central hyper-geometric distribution [23], which can be adjusted for gene length bias in DEGs.

#### KEGG pathway enrichment analysis

KEGG [24] is a database resource for understanding high-level functions and utilities of the biological system, such as the cell, the organism and the ecosystem, from molecular-level information, especially large-scale molecular datasets generated by genome sequencing and other high-throughput experimental technologies (http://www.genome.jp/kegg/). We used KOBAS [25] software to test the statistical enrichment of differential expression genes in KEGG pathways.

#### Validation of RNA-seq data by real-time quantitative PCR

To validate the expression of the candidate gene, real-time quantitative RT-PCR was employed by the method described previously by Chen et al. (2012, 2013) [26, 27], and the *CdACT2* gene was used as a quantitative control.

**Table 1:** Summary of sequence assembly after illumina sequencing

Sample name	Raw reads	Clean reads	Clean bases	Error rate (%)	Q20 (%)	Q30 (%)	GC content (%)
CdR_0	29891825	27957220	2.8G	0.04	96.21	88.26	51.29
CdS_0	29972660	27820617	2.78G	0.04	96.10	88.01	52.05
CdRCA_24	28507931	26729903	2.67G	0.04	96.55	89.09	53.10
CdSCA_24	32425088	30488049	3.05G	0.04	96.66	89.45	52.83
CdRCA_48	34416149	31852813	3.19G	0.06	94.65	85.70	52.91
CdSCA_48	35128459	32712066	3.27G	0.06	94.92	86.22	52.54
CdRNA_4	37227323	34328641	3.43G	0.06	94.83	86.16	52.21
CdSNA_4	42020195	39045618	3.9G	0.05	95.01	86.49	51.81
CdRCA_4	34145893	31628520	3.16G	0.04	94.78	86.06	52.84
CdSCA_4	29324652	27108530	2.71G	0.04	94.95	86.37	52.00

## Results

### Transcriptome sequencing and assembly

To comprehensively survey the genes associated with cold stress response in bermudagrass, ten cDNA libraries were constructed from total RNA extracted from leaves of bermudagrass (cold-resistant and cold-sensitive genotypes) with different cold treatments. The libraries were sequenced using the Illumina HiSeq™ 2000 platform. An overview of the RNA-Seq reads derived from the ten libraries was presented in Table 1. In total, 29,891,825, 28,507,931, 34,416,149, 34,145,893, 37,227,323 29,972,660, 32,425,088, 35,128,459, 29,324,652, 42,020,195 raw reads were generated in the CdR_0, CdRCA_24, CdRCA_48, CdRCA_4, CdRNA_4, CdS_0, CdSCA_24, CdSCA_48, CdSCA_4 and CdSNA_4, respectively (Table 1). To ensure the reliability of the libraries, we performed quality controls and obtained 27,957,220, 26,729,903, 31,852,813, 31,628,520, 34,328,641, 27,820,617, 30,488,049, 32,712,066, 27,108,530 and 39,045,618 clean reads for CdR_0, CdRCA_24, CdRCA_48, CdRCA_4, CdRNA_4, CdS_0, CdSCA_24, CdSCA_48, CdSCA_4 and CdSNA_4. Because of the absence of reference genomic sequences, de novo assembly was employed to construct transcripts from these RNA-seq reads. Trinity software was used for de novo assembly of the Illumina reads, which has been proven to be efficient for de novo reconstruction of transcriptomes from RNA-Seq data [21, 28]. A total of 326,435 contigs were obtained from the clean reads with a mean length of 1277 bp and length ranging from 201 bp to 20202 bp (Table 2). Among the 326,435 contigs, 121,166 unigenes were obtained with an average length of 706 bp. The longest and shortest unigene was 20,202 bp and 201 bp, respectively (N50 was 1276 bp, N90 was 269 bp).

**Table 2:** Length distribution of the transcripts and unigenes clustered from the de novo assembly

Category	Transcript	Unigene
200-500 bp	116004	79038
500-1kbp	61896	20515
1 k-2kbp	77618	11918
>2kbp	70917	9695
N50	2114	1276
N90	567	269
Total	326435	121166
Max length	20202	20202
Min length	201	201
Average length	1277	706

Note: The N50 size is computed by sorting all transcripts from largest to smallest and by determining the minimum set of transcripts whose sizes total 50 % of the entire transcript and unigene was the same; N90 was counted in the similar way

### Gene annotation

The unigenes were annotated by searching against the seven public databases (Table 3). The results showed that 35,679 unigenes (29.44 %) had significant matches in the Nr database, 25,662 (21.17 %) in the Nt database, 21,745 (17.94 %) in the Swiss-Prot database, 31,783 (26.23 %) in the GO database and 27,739 (22.89 %) in the PFAM database. In total, there were 43,945 unigenes (36.26 %) successfully annotated in at least one of the Nr, Nt, KO, Swiss-Prot, GO, KOG and Pfam databases, with 3999 unigenes (3.3 %) in all seven databases.

**Table 3:** The numbers and distribution rate of unigenes in the databases of NR, NT, KO, SWISS-PROT, PFAM, KOG and KEEG

	Number of Unigenes	Percentage (%)
Annotated in NR	35679	29.44
Annotated in NT	25662	21.17
Annotated in KO	7260	5.99
Annotated in SwissProt	21745	17.94
Annotated in PFAM	27739	22.89
Annotated in GO	31783	26.23
Annotated in KOG	10709	8.83
Annotated in all Databases	3999	3.3
Annotated in at least one Database	43945	36.26
Total Unigenes	121166	100

### Gene ontology (GO) classification

**Fig. 1:**
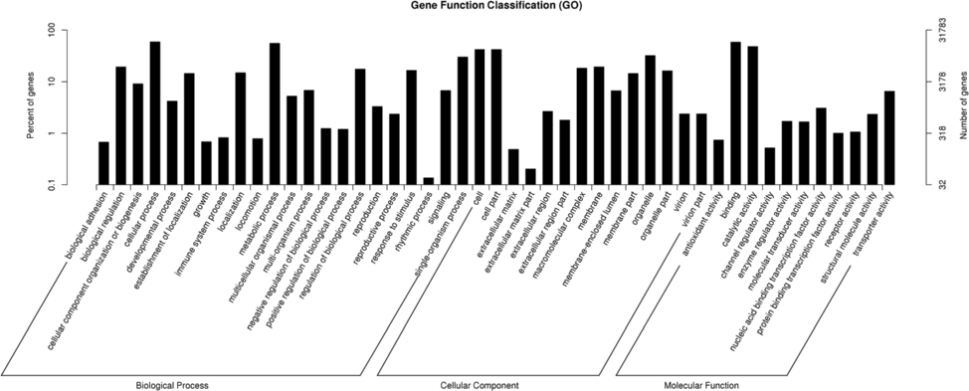
The numbers of DEGs identified in comparisons between pairs of libraries

For GO analysis, there were 31,783 unigenes divided into three ontologies (Fig. 1). For biological process (BP) category, genes involved in ‘cellular process’ (18,714), ‘metabolic process’ (17,627) and ‘single-organism process’(9506) were highly represented. The cellular component (CC)category mainly comprised proteins involved in ‘cell’ (13,324), ‘cell part’ (13,292) and ‘organelle’ (10,133). In terms of molecular function (MF) category, the highly represented molecular function was ‘binding’ (18,513), ‘catalytic activity’ (15,206) and ‘transporter activity’ (2076).

**Fig. 2:**
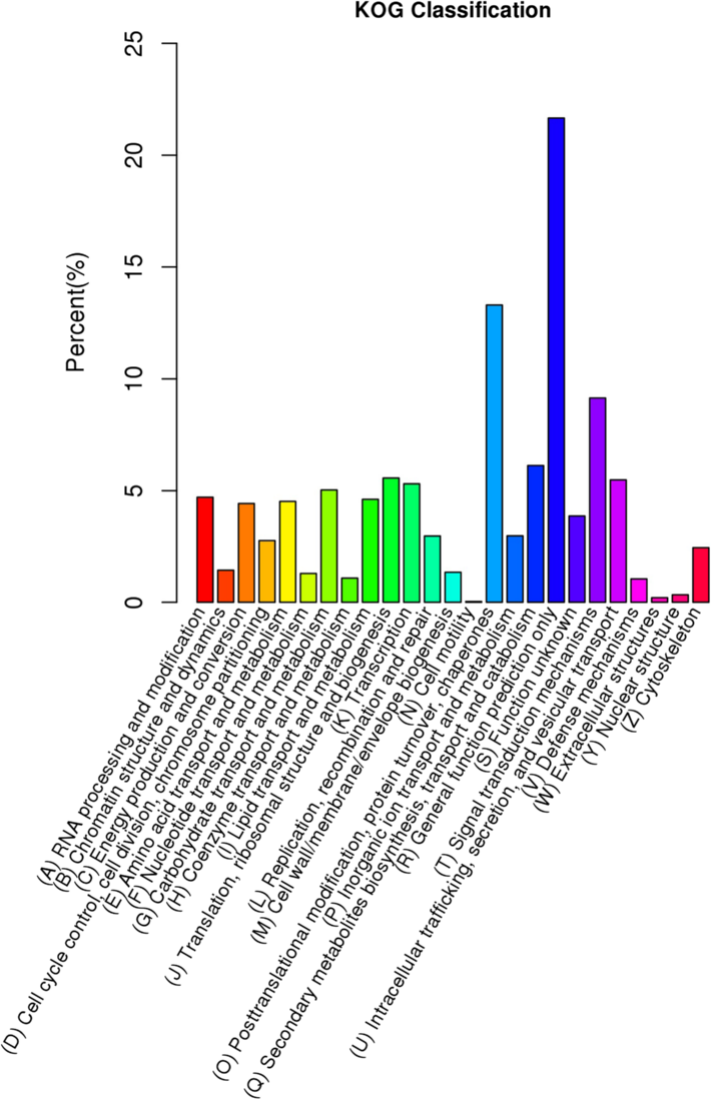
Histogram of gene ontology classification. The results are summarized in three main categories: biological process, cellular component and molecular function. The right y-axis indicates the number of genes in a category. The left y-axis indicates the percentage of a specific category of genes in that main category

In total, there were 10,709 unigenes assigned to KOG classification and divided into 25 specific categories (Fig. 2). The ‘general functional prediction only’ (2320) was the largest group, followed by ‘post-translational modification, protein turnover, chaperon’ (1424), ‘signal transduction mechanisms’ (979), ‘Secondary metabolites biosynthesis, transport and catabolism’(655), ‘Translation, ribosomal structure and biogenesis’ (595), ‘Intracellular trafficking and secretion, and vesicular transport’ (586). By contrast, only a few unigenes were assigned to ‘Cell motility’ (4) and ‘Extracellular structures’ (22).

**Fig. 3:**
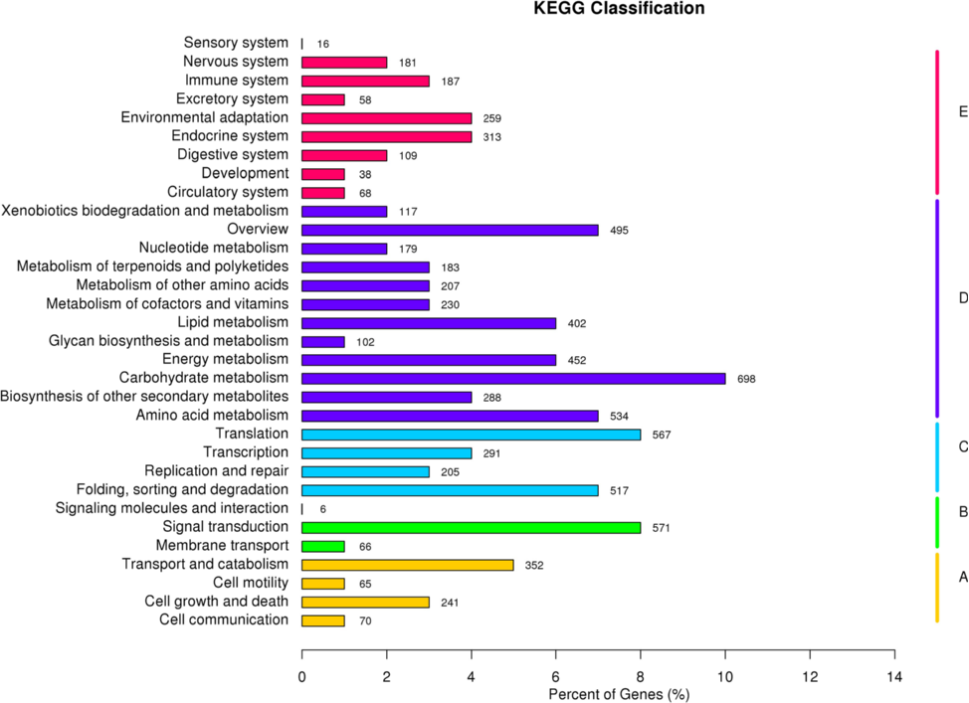
KOG annotation of putative proteins. In total, there were 10,709 unigenes assigned to KOG classification and divided into 25 specific categories. The x-axis indicates 25 groups of KOG. The y-axis indicates the percentage of the number of genes annotation under the group in the total number of genes annotation

The KEGG database is supposed to provide a systematic analysis of metabolic pathways and functions of gene products. To further identify the biological pathways that are active in bermudagrass, the 8067 unigenes annotated by blast analysis against KAAS (KEGG Automatic Annotation Server) were classified into five main biochemical pathways: ‘cellular processes’, ‘environmental information processing’, ‘genetic information processing’, ‘metabolism’ and ‘organismal systems’. The most represented pathways were ‘metabolism’ (3887 unigenes, 48.18 %) (Fig. 3). Among the 3887 unigenes in ‘metabolism’ pathway, ‘Carbohydrate metabolism’ (698), ‘Amino acid metabolism’ (534) ‘Energy metabolism’ (452) ‘Lipid metabolism’ (402) were highly represented (Fig. 3). The pathways related to ‘environmental information processing’ with the most representation were ‘signal transduction’ (571). These annotations provided a valuable resource for investigating the processes, functions, and pathways involved in cold response.

### Differential expression genes (DEGs) analysis under various cold treatments

**Fig. 4:**
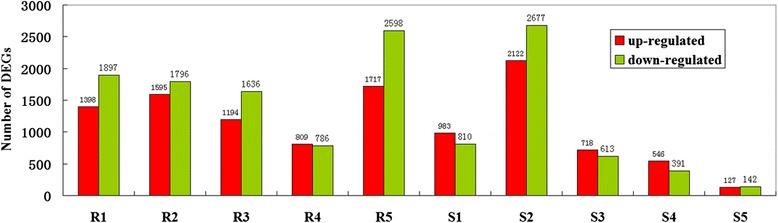
Functional classification and pathway assignment of unigenes by KEGG. The results are summarized in five main categories: A, Cellular Processes; B, Environmental Information Processing; C, Genetic Information Processing; D, Metabolism; E, Organismal Systems. The y-axis indicates the name of the KEGG metabolic pathways. The x-axis indicates the percentage of the number of genes annotation under the pathway in the total number of genes annotation

**Fig. 5:**
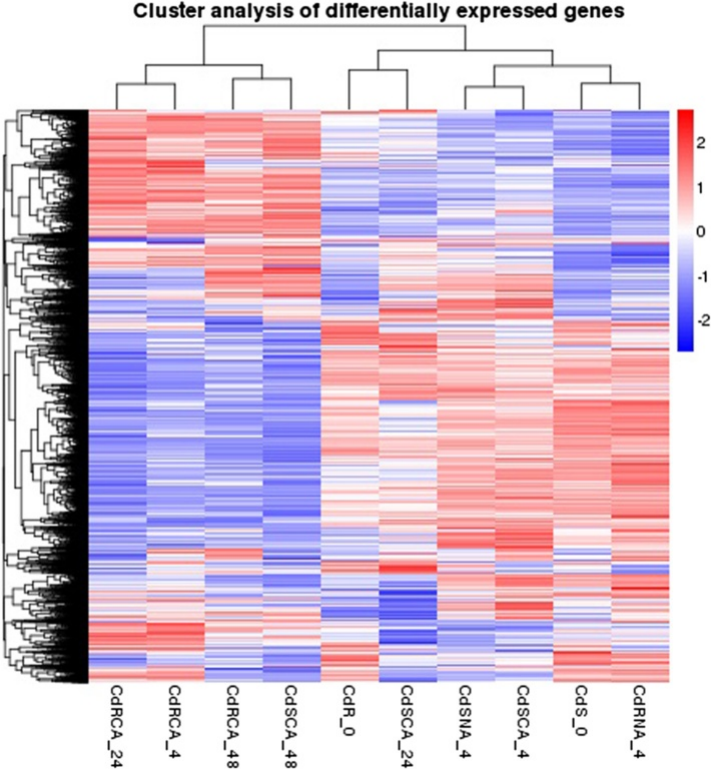
Hierarchical clustering of the differentially expressed genes

**Fig. 6:**
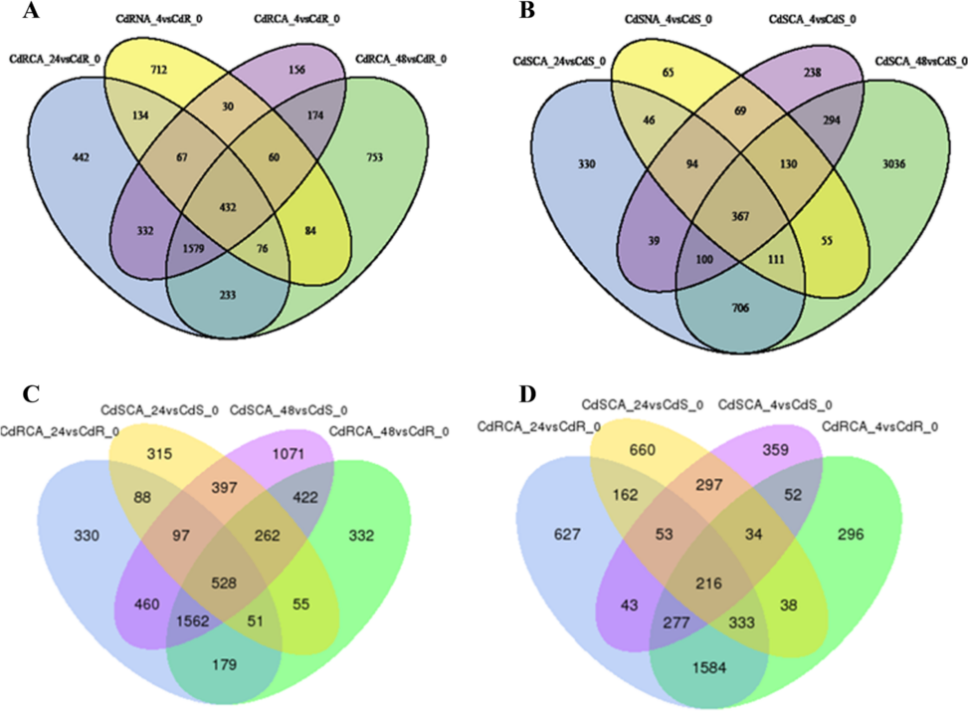
Venn diagram of differentially expressed genes. The sum of the numbers in each large circle represents total number of differentially expressed genes between comparison, the overlap part of the circles represents common differentially expressed genes between comparisons. **a** Four comparisons in R genotype (CdRCA_24 vs CdR_0; CdRCA_48 vs CdR_0; CdRCA_4 vs CdR_0; CdRNA_4 vs CdR_0); **b** Four comparisons in S genotype (CdSCA_24 vs CdS_0; CdSCA_48 vs CdS_0; CdSCA_4 vs CdS_0; CdSNA_4 vs CdS_0); **c** R and S genotypes have two comparisons (CdRCA_24 vs CdR_0; CdRCA_48 vs CdR_0; CdSCA_24 vs CdS_0; CdSCA_48 vs CdS_0), respectively. **d** R and S genotypes have two comparisons (CdRCA_24 vs CdR_0; CdRCA_4 vs CdR_0; CdSCA_24 vs CdS_0; CdSCA_4 vs CdS_0), respectively

DEGs (*q*-value < 0.005 and |log2 (fold change)| >1) were defined as genes that were significantly enriched or depleted in one sample relative to the other sample. From the ten comparisons, including treatment R1 (CdRCA_24 vs CdR_0), R2 (CdRCA_48 vs CdR_0), R3 (CdRCA_4 vs CdR_0), R4 (CdRNA_4 vs CdR_0), R5 (CdRCA_4 vs CdRNA_4), S1 (CdSCA_24 vs CdS_0), S2 (CdSCA_48 vs CdS_0), S3 (CdSCA_4 vs CdS_0), S4 (CdSNA_4 vs CdS_0) and S5 (CdSCA_4 vs CdSNA_4), the results showed that a large number of DEGs were identified. The number of DEGs detected was as follows: R1 3295 (1398 up- and 1897 down-regulated), R2 3391 ( 1595 up- and 1796 down-regulated), R3 2830 (1194 up- and 1636 down-regulated), R4 1595 (809 up- and 786 down-regulated), R5 4315 ( 1717 up- and 2598 down-regulated), S1 1793 (983 up- and 810 down-regulated), S2 4799 (2122 up- and 2677 down-regulated), S3 1331 (718 up- and 613 down-regulated), S4 937 (546 up- and 391 down-regulated) and S5 269 ( 127 up- and 142 down-regulated) (Fig. 4). Further hierarchical clustering method was employed to observe the overall expression pattern of the differentially expressed genes (Fig. 5). The blue bands identify low gene expression quantity, and the red represent the high gene expression quantity. The results revealed that more DEGs were detected in comparison R1 than that in S1, suggesting that global gene expressions were initiated more quickly in R genotype than those in S genotype, when they were exposed to cold stress. In addition, more DEGs were identified in the comparisons R3 and S3, which underwent a prior cold acclimation (CA) for 48 h, as compared to the treatments which didn’t undergo CA (R4 and S4), respectively (Figs. 4 and 5). It should be noted that the number of DEGs in R genotype is larger than that in S genotype, when they were subjected to freezing conditions (−5 °C for 4 h) with or without CA. However, there were no obvious differences between the comparisons S3 (CdSCA_4 vs CdS_0) and S4 (CdSNA_4 vs CdS_0) from the hierarchical clustering analysis (Fig. 5). When comparing the R5 (CdRCA_4 vs CdRNA_4) and S5 (CdSCA_4 vs CdSNA_4) treatments, it was surprisingly found that R5 had 4315 DEGs (1717 up- and 2598 down-regulated), whereas only 269 DEGs (127 up- and 142 down-regulated) were identified in S5 treatment. Further analysis using a venn diagram showed that both unique and overlapping sets of differentially expressed genes were detected at each treatment in both R and S genotypes (Fig. 6). Among these DEGs, 432 were categorized as commonly induced genes in R genotype comparisons, R1 (CdRCA_24 vs CdR_0), R2 (CdRCA_48 vs CdR_0), R3 (CdRCA_4 vs CdR_0) and R4 (CdRNA_4 vs CdR_0), while 367 were identified as overlap in four S genotype comparisons, S1 (CdSCA_24 vs CdS_0), S2 (CdSCA_48 vs CdS_0), S3 (CdSCA_4 vs CdS_0) and S4 (CdSNA_4 vs CdS_0) (Fig. 6).

### GO classification of differentially transcribed genes

**Fig. 7:**
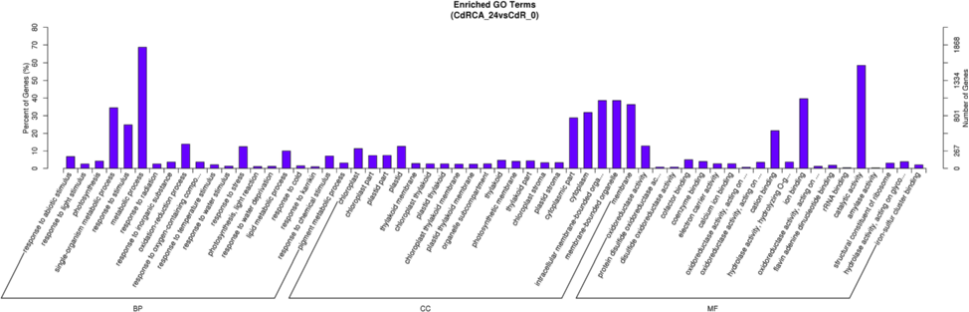
Gene Ontology (GO) classification of the DEGs identified in R1 comparison between a pair of libraries. DEGs were annotated in three categories: biological process, cellular component and molecular function. Y-axis (right) represents the number of DEGs in each category; Y-axis (left) represents the percentage of a specific category of DEGs within that main category

In the treatment R1, 2669 of the 3295 DEGs could be assigned as a GO term. The equivalent number for other comparisons were as follows: treatment R2, 2722/3391; R3, 2313/2830; R4, 1317/1595; R5, 3592/4315; S1, 1439/1793; S2, 3920/4799; S3, 1017/1331; S4, 717/ 937; S5, 214/ 269 (Additional file 2). For DEG enriched GO classification in the R1 comparison, 20 GO classes fell into the categories “biological process”, 20 into “cellular component” and 20 into “molecular function” (Fig. 7). The equivalent distribution in R2 was 20, 20 and 7; in R3 was 20, 20 and 20; in R4 was 20, 20 and 14; in S1 was 20, 20 and 4; in S2 was 20, 20 and 20; in S3 was 7, 8 and 2; in S4 was 6, 6 and 0 (Additional file 3). The major classes of biological process among the DEGs in the R1 comparison were “metabolic process”, “single-organism metabolic process”, “response to stimulus”, “oxidation-reduction process”, “response to stress”, “lipid metabolic process” and “response to abiotic stimulus”; the predominant cellular components were “membrane-bounded organelle”, “intracellular membrane-bounded organelle”, “membrane”, “cytoplasm”, “cytoplasmic part”, “plastid”, and “chloroplast”; and for molecular function “catalytic activity”, “ion binding”, “cation binding” and “oxidoreductase activity” (Fig. 7). The details of GO classification of DEGs in other comparisons are shown in Additional file 3.

### Function annotation of DEGs using the KEGG database

Unigene KEGG annotation was aimed at DEGs from the above comparisons. In the R1 comparison, 1531 DEGs were assigned to the KEGG database involving 160 pathways; for R2, 1413 DEGs were assigned to 159 pathways; for R3, 1245 DEGs were assigned to 156 pathways; for R4, 914 DEGs were assigned to 125 pathways; for S1, 948 DEGs were assigned to 138 pathways; for S2, 2345 DEGs were assigned to 167 pathways; for S3, 510 DEGs were assigned to 118 pathways; and for S4, 461 DEGs were assigned to 120 pathways. The details of the KEGG classification of the above comparisons are presented in Additional file 4.

### Genes involved in the response to low temperature

The Ca^2+^ signaling components mainly included calcium-binding protein (CBP), calmodulin-like protein (CML), calcium-dependent protein kinase (CDPK), calcineurin B-like protein (CBL), CBL-interacting protein kinases (CIPK), and calmodulin-binding receptor like kinases (CBRLK) [29]. In the R1 comparison, there were 6 CML, 2 CBRLK, 3 calmodulin-binding protein, 2 Calcium-binding protein, 1 extracellular calcium sensing receptor, 3 CDPK, 1 CBL and 12 CIPK. The equivalent order for the R2 comparison was, respectively, one, four, three, two, one, three, one and nineteen; for R3 comparison, two, three, one, two, one, three, one and eleven; for R4 comparison, four, zero, two, one, one, zero, one and four; for S1 comparison, three, zero, two, two, zero, two, zero and seven; for S2 comparison, ten, three, six, one, one, three, one and thirteen; for S3 comparison, zero, one, one, two, zero, three, zero and four; for S4 comparison, there are only one CBP, one CDPK and three CIPK. Among these differential expression Ca^2+^ signaling genes, the expression of unigene (comp148141_c0) encoding calcium binding protein was up-regulated in the comparisons R2, R3, R4, S1 and S3. The transcripts of CBP unigene (comp132952_c0) was induced in R2, S1, S2, S3 and S4. By contrast, another *CBP* gene expression was only induced in the cold-resistant bermudagrass genotype under cold treatment (comparisons R1 and R3). It is very interesting to find that one gene expression (comp151017_c0) encoding extracellular calcium sensing receptor was up-regulated in comparisons R1, R2, R3 and S2, but down-regulated in R4. One *CDPK* gene (comp156791_c0) transcripts were also accumulated in comparisons R1, R2, R3 and S2. The complete details of DEGs involved in Ca^2+^ signalling pathway are presented in Additional file 5. The CBL–CIPK signaling networks have been proven to play important roles in response to a wide range of stimuli. Here, only two *CBL* genes were identified as DEGs, and both genes were up-regulated by cold treatment. Induction of expression of one *CBL* gene (comp151010_c0) was observed in the following comparisons R1, R2, R3 and S2. Besides, another one *CBL* gene (comp151988_c1) was induced in comparisons R4, showing that the gene may be involved in plant response to chilling stress without a prior CA. The number of differentially expressed *CIPK* genes was 46 and 27 in comparisons of cold-resistant and –sensitive genotypes of bermudagrass, respectively, revealing that more *CIPK* genes were involved in cold response in the cold-resistant genotype. It was interestingly found that most of the identified *CIPK* genes were down-regulated by cold stress, while 7 genes identified in the S1 comparison were all up-regulated. These results revealed that expression profiles of *CIPK* genes were different in R and S genotypes under cold condition. The complete details of DEGs associated with CIPK are presented in Additional file 6. Similarly, DEGs associated with the MAPK cascade were twelve in comparisons of cold-resistant genotype, while only seven related genes were detected in comparisons of cold-sensitive genotype. The complete details of DEGs associated with MAPK are presented in Table 4. One MAPKKK gene (comp155944_c1) was found to be down-regulated in R1, R2, R3 and S3 comparisons, implying that the gene may be specifically involved in the CA process. The expression of another *MAPKKK* gene (comp158986_c0) was induced in R2, R3 and R4 comparisons, and the induction folds were higher in R3 (5.26) than that in R4 (3.51) comparisons, suggesting that the gene could be more effectively activated to respond to chilling treatment after CA process.

**Table 4:** The differential gene expression of *MAPK* genes in each comparison

Comparison	GeneID	Log2 ratio	Up-down regulation	P-value	q-value	Gene description
R1	Comp155918_c0	−2.5726	Down	1.62E-16	7.06E-15	Mitogen-activated protein kinase 5 OS = Oryza sativa subsp. japonica
	Comp155944_c1	−2.4339	Down	3.68E-71	6.94E-69	Mitogen-activated protein kinase kinase kinase 2 OS = Arabidopsis thaliana
R2	Comp154749_c0	3.9782	Up	4.83E-19	2.52E-17	Probable serine/threonine-protein kinase DDB_G0278901 OS = Dictyostelium discoideum GN = DDB_G0278901 PE = 3 SV = 1
	Comp155944_c1	−2.0886	Down	5.83E-58	9.50E-56	Mitogen-activated protein kinase kinase kinase 2 OS = Arabidopsis thaliana GN = ANP2 PE = 2 SV = 1
	Comp158986_c0	6.0635	Up	8.84E-34	7.84E-32	Mitogen-activated protein kinase kinase kinase A OS = Dictyostelium discoideum GN = mkkA PE = 1 SV = 2
R3	Comp154749_c0	3.5763	Up	8.12E-17	4.90E-15	Probable serine/threonine-protein kinase DDB_G0278901 OS = Dictyostelium discoideum GN = DDB_G0278901 PE = 3 SV = 1
	Comp155918_c0	−1.2133	Down	3.11E-05	0.000661	Mitogen-activated protein kinase 5 OS = Oryza sativa subsp. japonica GN = MPK5 PE = 1 SV = 1
	Comp155944_c1	−2.4658	Down	1.53E-62	3.38E-60	Mitogen-activated protein kinase kinase kinase 2 OS = Arabidopsis thaliana GN = ANP2 PE = 2 SV = 1
	Comp158986_c0	5.2605	Up	1.87E-23	1.51E-21	Mitogen-activated protein kinase kinase kinase A OS = Dictyostelium discoideum GN = mkkA PE = 1 SV = 2
R4	Comp155918_c0	2.5994	Up	2.24E-113	2.94E-110	Mitogen-activated protein kinase 5 OS = Oryza sativa subsp. japonica GN = MPK5 PE = 1 SV = 1
	Comp156595_c0	−2.1996	Down	6.93E-60	3.34E-57	Mitogen-activated protein kinase 14 OS = Oryza sativa subsp. japonica GN = MPK14 PE = 2 SV = 1
	Comp158986_c0	3.5159	Up	3.67E-07	1.62E-05	Mitogen-activated protein kinase kinase kinase A OS = Dictyostelium discoideum GN = mkkA PE = 1SV = 2
S1	Comp153907_c0	2.5075	Up	3.72E-27	6.29E-25	Mitogen-activated protein kinase 4 OS = Oryza sativa subsp. japonica GN = MPK4 PE = 2 SV = 1
	Comp156595_c0	1.3245	Up	2.81E-24	4.12E-22	Mitogen-activated protein kinase 14 OS = Oryza sativa subsp. japonica GN = MPK14 PE = 2 SV = 1
S2	Comp153907_c0	1.2575	Up	0.0002751	0.0025652	Mitogen-activated protein kinase 4 OS = Oryza sativa subsp. japonica GN = MPK4 PE = 2 SV = 1
	Comp155944_c1	−1.8891	Down	1.99E-51	1.73E-49	Mitogen-activated protein kinase kinase kinase 2 OS = Arabidopsis thaliana GN = ANP2 PE = 2 SV = 1
S3	Comp153907_c0	1.5408	Up	1.36E-15	1.83E-13	Mitogen-activated protein kinase 4 OS = Oryza sativa subsp. japonica GN = MPK4 PE = 2 SV = 1
	Comp156595_c0	−1.0632	Down	3.72E-10	3.06E-08	Mitogen-activated protein kinase 14 OS = Oryza sativa subsp. japonica GN = MPK14 PE = 2 SV = 1
S4	Comp153907_c0	1.7287	Up	6.67E-15	1.11E-12	Mitogen-activated protein kinase 4 OS = Oryza sativa subsp. japonica GN = MPK4 PE = 2 SV = 1

In the present study, members of various low temperature-responsive transcription factor (TF) families were identified. The major TF families presented were AP2/ERF, bHLH, WRKY and NAC family. There are 7 and 6 cold up-regulated genes associated with the NAC family identified in various comparisons in R and S genotypes, respectively (Table 5). Of these *NAC* genes, comp148886_c0 and comp150085_c0 were induced by low temperature in both R and S genotypes, but the induction folds by cold were higher in R genotype than that in S genotype.

**Table 5:** The up-regulated expression of NAC TFs in R and S genotype

Genotype	GeneID	Log2 ratio	P-value	q-value	Gene description
R	Comp132790_c1	1.9536	6.37E-05	0.00129	NAC transcription factor ONAC010 OS = Oryza sativa subsp. japonica GN = ONAC010 PE = 2 SV = 1
	Comp146262_c0	1.6583	1.41E-06	5.74E-05	NAC transcription factor 1 [Bambusa emeiensis]
	Comp150085_c0	3.5182	1.65E-18	2.06E-16	NAC domain-containing protein 48 OS = Oryza sativa subsp. japonica GN = NAC48 PE = 2 SV = 1
	Comp156007_c0	1.0682	9.27E-11	6.34E-09	NAC domain-containing protein 74 OS = Oryza sativa subsp. japonica GN = NAC74 PE = 2 SV = 1
	Comp156333_c0	1.7897	1.41E-18	1.77E-16	NAC transcription factor NAM-B2 OS = Triticum durum GN = NAM-B2 PE = 2 SV = 1
	Ccomp148886_c0	2.2164	2.37E-60	1.17E-57	NAC domain-containing protein 67 OS = Oryza sativa subsp. japonica GN = NAC67 PE = 2 SV = 1
	Comp150085_c0	3.5182	1.65E-18	2.06E-16	NAC domain-containing protein 48 OS = Oryza sativa subsp. japonica GN = NAC48 PE = 2 SV = 1
S	Comp150613_c0	1.9079	4.56E-98	5.77E-95	NAC domain-containing protein 71 OS = Oryza sativa subsp. japonica GN = NAC71 PE = 2 SV = 1
	Comp139455_c0	3.0897	1.48E-31	3.05E-29	NAC domain-containing protein 67 OS = Oryza sativa subsp. japonica GN = NAC67 PE = 2 SV = 1
	Comp155476_c0	1.7018	1.36E-13	3.36E-12	NAC domain-containing protein 21/22 OS = Arabidopsis thaliana GN = NAC021 PE = 1 SV = 2
	Comp148886_c0	1.3914	1.29E-37	5.82E-35	NAC domain-containing protein 67 OS = Oryza sativa subsp. japonica GN = NAC67 PE = 2 SV = 1
	Comp150085_c0	1.4897	2.56E-05	0.000933	NAC domain-containing protein 48 OS = Oryza sativa subsp. japonica GN = NAC48 PE = 2 SV = 1
	Comp155462_c1	1.2659	4.28E-80	7.36E-77	NAC domain-containing protein 48 OS = Oryza sativa subsp. japonica GN = NAC48 PE = 2 SV = 1

As shown in Table 6, comp160681_c0 and comp160771_c0 encoding WRKY TF were up-regulated in the R1, R2, R3 and S2 comparisons, suggesting that these two WRKY proteins are involved in the CA process in both R and S genotypes, but specifically involved in response to freezing treatment in plants with prior exposure to CA in R genotype. Another *WRKY* gene (comp160978_c0) was found to be differentially transcribed in R2, R3, R4, S1 and S3, implying that it is involved in the CA process and freezing treatment in plants with prior exposure to CA in both R and S genotypes, and that this *WRKY* gene also plays essential roles in freezing treatment without prior CA process in the R genotype. Moreover, comp144527_c0 gene was differentially expressed in R2, R3, S1 and S3 comparisons.

**Table 6:** The up-regulated expression of WRKY TFs in each comparison

Genotype	GeneID	log2 ratio	P-value	q-value	Gene description
R1	Comp160681_c0	1.5684	2.72E-06	5.18E-05	Probable WRKY transcription factor 2 OS = Arabidopsis thaliana GN = WRKY2 PE = 2 SV = 1
	Comp160771_c0	2.6429	5.54E-06	0.000101	Probable WRKY transcription factor 19 OS = Arabidopsis thaliana GN = WRKY19 PE = 2 SV = 1
R2	Comp144527_c0	5.541	1.57E-26	1.11E-24	Probable WRKY transcription factor 53 OS = Arabidopsis thaliana GN = WRKY53 PE = 1 SV = 1
	Comp147411_c0	1.5419	1.21E-37	1.20E-35	Probable WRKY transcription factor 70 OS = Arabidopsis thaliana GN = WRKY70 PE = 2 SV = 1
	Comp152445_c0	3.577	1.71E-22	1.06E-20	WRKY-type transcription factor, partial [Zea mays subsp. mays]
	Comp155955_c0	1.5367	3.30E-38	3.35E-36	Probable WRKY transcription factor 40 OS = Arabidopsis thaliana GN = WRKY40 PE = 1 SV = 1
	Comp160681_c0	1.6972	3.81E-08	9.28E-07	Probable WRKY transcription factor 2 OS = Arabidopsis thaliana GN = WRKY2 PE = 2 SV = 1
	Comp160771_c0	2.3539	6.71E-05	0.001025	Probable WRKY transcription factor 19 OS = Arabidopsis thaliana GN = WRKY19 PE = 2 SV = 1
	Comp160978_c0	4.5088	8.95E-141	4.67E-138	Probable WRKY transcription factor 41 OS = Arabidopsis thaliana GN = WRKY41 PE = 2 SV = 2
R3	Comp144527_c0	3.8106	1.81E-09	6.57E-08	Probable WRKY transcription factor 53 OS = Arabidopsis thaliana GN = WRKY53 PE = 1 SV = 1
	Comp160681_c0	1.3662	2.90E-07	8.24E-06	Probable WRKY transcription factor 2 OS = Arabidopsis thaliana GN = WRKY2 PE = 2 SV = 1
	Comp160771_c0	2.0982	7.57E-05	0.001518	Probable WRKY transcription factor 19 OS = Arabidopsis thaliana GN = WRKY19 PE = 2 SV = 1
	Comp160978_c0	2.2264	5.15E-22	3.98E-20	Probable WRKY transcription factor 41 OS = Arabidopsis thaliana GN = WRKY41 PE = 2 SV = 2
R4	Comp140243_c0	2.7427	2.06E-09	1.24E-07	TPA: putative WRKY DNA-binding domain superfamily protein [Zea mays]
	Comp141474_c0	3.0433	6.18E-08	3.06E-06	Probable WRKY transcription factor 33 OS = Arabidopsis thaliana GN = WRKY33 PE = 1 SV = 2
	Comp143904_c0	3.0347	8.47E-09	4.77E-07	putative WRKY DNA-binding domain superfamily protein [Zea mays]
	Comp153544_c0	1.1647	1.21E-07	5.74E-06	Probable WRKY transcription factor 46 OS = Arabidopsis thaliana GN = WRKY46 PE = 2 SV = 1
	Comp155955_c0	1.3985	3.92E-47	1.39E-44	Probable WRKY transcription factor 40 OS = Arabidopsis thaliana GN = WRKY40 PE = 1 SV = 1
	Comp157039_c0	1.0313	1.69E-07	7.87E-06	Probable WRKY transcription factor 19 OS = Arabidopsis thaliana GN = WRKY19 PE = 2 SV = 1
	Comp160978_c0	1.6743	4.81E-12	3.73E-10	Probable WRKY transcription factor 41 OS = Arabidopsis thaliana GN = WRKY41 PE = 2 SV = 2
	Comp161665_c1	5.3647	1.76E-12	1.41E-10	Probable WRKY transcription factor 30 OS = Arabidopsis thaliana GN = WRKY30 PE = 2 SV = 1
S1	Comp144527_c0	4.244	7.03E-05	0.0016062	Probable WRKY transcription factor 53 OS = Arabidopsis thaliana GN = WRKY53 PE = 1 SV = 1
	Comp144804_c0	2.7015	0.0001009	0.0022066	WRKY transcription factor 18 OS = Arabidopsis thaliana GN = WRKY18 PE = 1 SV = 2
	Comp147411_c0	1.3895	4.39E-06	0.0001321	Probable WRKY transcription factor 70 OS = Arabidopsis thaliana GN = WRKY70 PE = 2 SV = 1
	Comp151582_c0	2.1332	1.25E-11	7.95E-10	Probable WRKY transcription factor 51 OS = Arabidopsis thaliana GN = WRKY51 PE = 2 SV = 1
	Comp151601_c0	2.3956	2.39E-13	1.80E-11	Probable WRKY transcription factor 11 OS = Arabidopsis thaliana GN = WRKY11 PE = 2 SV = 2
	Comp152382_c0	1.4257	1.85E-43	5.63E-41	Probable WRKY transcription factor 26 OS = Arabidopsis thaliana GN = WRKY26 PE = 2 SV = 2
	Comp152445_c0	3.3658	1.21E-09	6.29E-08	WRKY-type transcription factor, partial [Zea mays subsp. mays]
	Comp155955_c0	1.3893	2.16E-22	2.89E-20	Probable WRKY transcription factor 40 OS = Arabidopsis thaliana GN = WRKY40 PE = 1 SV = 1
	Comp160893_c0	2.7219	2.32E-05	0.0005938	Probable WRKY transcription factor 11 OS = Arabidopsis thaliana GN = WRKY11 PE = 2 SV = 2
	Comp160978_c0	1.5478	0.0001598	0.0033056	Probable WRKY transcription factor 41 OS = Arabidopsis thaliana GN = WRKY41 PE = 2 SV = 2
	Comp161539_c0	1.1412	1.99E-36	4.72E-34	Probable WRKY transcription factor 19 OS = Arabidopsis thaliana GN = WRKY19 PE = 2 SV = 1
S2	Comp152445_c0	3.6232	8.24E-12	1.80E-10	WRKY-type transcription factor, partial [Zea mays subsp. mays]
	Comp159675_c0	2.2696	6.13E-13	1.45E-11	Probable WRKY transcription factor 34 OS = Arabidopsis thaliana GN = WRKY34 PE = 2 SV = 1
	Comp160681_c0	1.4438	0.0001106	0.0011127	Probable WRKY transcription factor 2 OS = Arabidopsis thaliana GN = WRKY2 PE = 2 SV = 1
	Comp160771_c0	2.8677	1.71E-05	0.0001977	Probable WRKY transcription factor 19 OS = Arabidopsis thaliana GN = WRKY19 PE = 2 SV = 1
S3	Comp144527_c0	3.9213	1.50E-05	0.0005733	Probable WRKY transcription factor 53 OS = Arabidopsis thaliana GN = WRKY53 PE = 1 SV = 1
	Comp151582_c0	1.343	3.15E-08	1.97E-06	Probable WRKY transcription factor 51 OS = Arabidopsis thaliana GN = WRKY51 PE = 2 SV = 1
	Comp151601_c0	1.4217	1.04E-07	5.94E-06	Probable WRKY transcription factor 11 OS = Arabidopsis thaliana GN = WRKY11 PE = 2 SV = 2
	Comp160893_c0	2.2538	1.20E-05	0.0004685	Probable WRKY transcription factor 11 OS = Arabidopsis thaliana GN = WRKY11 PE = 2 SV = 2
	Comp160978_c0	2.1188	5.16E-18	8.10E-16	Probable WRKY transcription factor 41 OS = Arabidopsis thaliana GN = WRKY41 PE = 2 SV = 2
	Comp161665_c1	4.0455	3.45E-05	0.0012143	Probable WRKY transcription factor 30 OS = Arabidopsis thaliana GN = WRKY30 PE = 2 SV = 1

There are seven, ten, six and three up-regulated expressed genes encoding bHLH transcription factors identified in R1, R2, R3 and R4 comparisons of R genotype, respectively, whereas four, ten, two and two were detected in S1, S2, S3 and S4 comparisons of the S genotype (Table 7). Overall, the number of up-regulated expressed *bHLH* genes in R genotype was greater than that of S genotype. Of the identified *bHLH* genes, the log2 (fold change) of comp155113_c1 gene reached to 6.4 and 4.98 in R2 and S2 comparisons, respectively, but it was not detected as DEGs in other comparisons. In addition, the *bHLH* gene (comp151458_c0) expression was largely up-regulated in R1, R2 and R3 comparisons, while it was only found to be increased in the S2 comparison (Table 7).

**Table 7:** The up-regulated expression of bHLH TFs in each comparison

Genotype	GeneID	log2 ratio	*P*-value	*q*-value	Gene description
R1	Comp150821_c0	1.0287	0.0001158	0.0017186	Transcription factor ILR3 OS = Arabidopsis thaliana GN = ILR3 PE = 1 SV = 1
	Comp157296_c0	1.3757	1.45E-25	9.54E-24	Transcription factor PIF5 OS = Arabidopsis thaliana GN = PIF5 PE = 1 SV = 1
	Comp156748_c0	1.4473	8.95E-07	1.82E-05	Transcription factor bHLH49 OS = Arabidopsis thaliana GN = BHLH49 PE = 2 SV = 1
	Comp152649_c0	4.6376	5.91E-14	2.23E-12	Transcription factor bHLH47 OS = Arabidopsis thaliana GN = BHLH47 PE = 2 SV = 1
	Comp151458_c0	4.8684	5.49E-93	1.38E-90	Transcription factor ORG2 OS = Arabidopsis thaliana GN = ORG2 PE = 1 SV = 1
	Comp155000_c0	1.8371	4.24E-22	2.47E-20	Transcription factor bHLH140 OS = Arabidopsis thaliana GN = BHLH140 PE = 4 SV = 1
	Comp152649_c0	4.6376	5.91E-14	2.23E-12	Transcription factor bHLH47 OS = Arabidopsis thaliana GN = BHLH47 PE = 2 SV = 1
R2	Comp150821_c0	1.0589	1.49E-05	0.0002559	Transcription factor ILR3 OS = Arabidopsis thaliana GN = ILR3 PE = 1 SV = 1
	Comp151458_c0	5.295	2.09E-133	1.03E-130	Transcription factor ORG2 OS = Arabidopsis thaliana GN = ORG2 PE = 1 SV = 1
	Comp152402_c0	2.7546	7.47E-25	5.06E-23	Transcription factor bHLH92 OS = Arabidopsis thaliana GN = BHLH92 PE = 2 SV = 1
	Comp152649_c0	4.3428	7.24E-12	2.46E-10	Transcription factor bHLH47 OS = Arabidopsis thaliana GN = BHLH47 PE = 2 SV = 1
	Comp155000_c0	1.3155	4.75E-11	1.52E-09	Transcription factor bHLH140 OS = Arabidopsis thaliana GN = BHLH140 PE = 4 SV = 1
	Comp155113_c1	6.4166	4.35E-06	8.08E-05	Transcription factor bHLH100 OS = Arabidopsis thaliana GN = BHLH100 PE = 2 SV = 1
	Comp156748_c0	1.7023	1.19E-10	3.70E-09	Transcription factor bHLH49 OS = Arabidopsis thaliana GN = BHLH49 PE = 2 SV = 1
	Comp157296_c0	1.6831	3.05E-47	3.96E-45	Transcription factor PIF5 OS = Arabidopsis thaliana GN = PIF5 PE = 1 SV = 1
	Comp158090_c0	2.7887	1.36E-05	0.000235	Transcription factor bHLH87 OS = Arabidopsis thaliana GN = BHLH87 PE = 2 SV = 1
	Comp161381_c0	1.7318	4.00E-18	1.99E-16	Transcription factor MYC4 OS = Arabidopsis thaliana GN = BHLH4 PE = 2 SV = 1
R3	Comp151458_c0	5.1734	1.12E-145	6.96E-143	Transcription factor ORG2 OS = Arabidopsis thaliana GN = ORG2 PE = 1 SV = 1
	Comp152649_c0	3.9009	2.63E-10	1.03E-08	Transcription factor bHLH47 OS = Arabidopsis thaliana GN = BHLH47 PE = 2 SV = 1
	Comp155000_c0	1.8218	1.13E-32	1.28E-30	Transcription factor bHLH140 OS = Arabidopsis thaliana GN = BHLH140 PE = 4 SV = 1
	Comp158090_c0	2.3542	8.95E-05	0.0017648	Transcription factor bHLH87 OS = Arabidopsis thaliana GN = BHLH87 PE = 2 SV = 1
	Comp157296_c0	1.3594	8.99E-42	1.32E-39	Transcription factor PIF5 OS = Arabidopsis thaliana GN = PIF5 PE = 1 SV = 1
R4	Comp144776_c0	4.2436	2.26E-08	1.20E-06	putative HLH DNA-binding domain superfamily protein [Zea mays]
	Comp152402_c0	2.1585	9.87E-18	1.17E-15	Transcription factor bHLH92 OS = Arabidopsis thaliana GN = BHLH92 PE = 2 SV = 1
	Comp161008_c0	1.0341	2.20E-09	1.31E-07	Transcription factor bHLH62 OS = Arabidopsis thaliana GN = BHLH62 PE = 2 SV = 1
S1	Comp152402_c0	5.684	2.77E-50	1.01E-47	Transcription factor bHLH92 OS = Arabidopsis thaliana GN = BHLH92 PE = 2 SV = 1
	Comp152681_c0	2.2077	2.22E-24	3.30E-22	Transcription factor bHLH113 OS = Arabidopsis thaliana GN = BHLH113 PE = 2 SV = 1
	Comp153254_c1	2.4778	2.26E-07	8.65E-06	Transcription factor bHLH35 OS = Arabidopsis thaliana GN = BHLH35 PE = 2 SV = 1
	Comp161381_c0	1.8375	5.99E-18	6.18E-16	Transcription factor MYC4 OS = Arabidopsis thaliana GN = BHLH4 PE = 2 SV = 1
S2	Comp150821_c0	1.9992	1.56E-10	3.08E-09	Transcription factor ILR3 OS = Arabidopsis thaliana GN = ILR3 PE = 1 SV = 1
	Comp151458_c0	6.2918	2.52E-122	5.90E-120	Transcription factor ORG2 OS = Arabidopsis thaliana GN = ORG2 PE = 1 SV = 1
	Comp152649_c0	4.7211	7.00E-29	3.47E-27	Transcription factor bHLH47 OS = Arabidopsis thaliana GN = BHLH47 PE = 2 SV = 1
	Comp153254_c1	2.4642	3.92E-07	5.64E-06	Transcription factor bHLH35 OS = Arabidopsis thaliana GN = BHLH35 PE = 2 SV = 1
	Comp155000_c0	2.3306	1.24E-21	4.69E-20	Transcription factor bHLH140 OS = Arabidopsis thaliana GN = BHLH140 PE = 4 SV = 1
	Comp155113_c1	4.9895	0.0001235	0.001231	Transcription factor bHLH100 OS = Arabidopsis thaliana GN = BHLH100 PE = 2 SV = 1
	Comp156748_c0	2.3306	7.63E-17	2.27E-15	Transcription factor bHLH49 OS = Arabidopsis thaliana GN = BHLH49 PE = 2 SV = 1
	Comp157296_c0	1.5876	1.65E-21	6.21E-20	Transcription factor PIF5 OS = Arabidopsis thaliana GN = PIF5 PE = 1 SV = 1
	Comp158090_c0	3.0266	1.90E-06	2.52E-05	Transcription factor bHLH87 OS = Arabidopsis thaliana GN = BHLH87 PE = 2 SV = 1
	Comp161381_c0	1.1771	2.24E-05	0.0002535	Transcription factor MYC4 OS = Arabidopsis thaliana GN = BHLH4 PE = 2 SV = 1
S3	Comp152402_c0	3.652	1.49E-16	2.14E-14	Transcription factor bHLH92 OS = Arabidopsis thaliana GN = BHLH92 PE = 2 SV = 1
	Comp161381_c0	1.7077	1.87E-31	6.20E-29	Transcription factor MYC4 OS = Arabidopsis thaliana GN = BHLH4 PE = 2 SV = 1
S4	Comp152681_c0	1.6033	3.85E-16	7.24E-14	Transcription factor bHLH113 OS = Arabidopsis thaliana GN = BHLH113 PE = 2 SV = 1
	Comp161381_c0	1.1696	6.53E-10	6.46E-08	Transcription factor MYC4 OS = Arabidopsis thaliana GN = BHLH4 PE = 2 SV = 1

CBF TFs belong to the AP2/ERF (APETALA2/ethylene-responsive factor) superfamily. In our present study, comp133037_c0 gene encoding CBF3 TFs was found to be specifically and highly expressed in R genotype (R2 and R3 comparisons), and the log2 folds were increased to 7.04 and 5.99, respectively. Two *CBF* genes (comp143318_c0 and comp155879_c0) were commonly expressed in R and S genotypes (Table 8). In our present study, we identified low temperature sensing and signaling related genes and transcription factors as DEGs under different cold treatments. In addition, various functional proteins, such as LEA proteins and dehydrins, also accumulated under cold conditions.

**Table 8:** The up-regulated expression of CBF TFs in each comparison

Genotype	GeneID	log2 ratio	P-value	q-value	Gene description
R2	Comp133037_c0	7.0454	1.82E-08	4.59E-07	Dehydration-responsive element-binding protein 1A OS = Oryza sativa subsp. japonica GN = DREB1A PE = 2 SV = 1
	Comp143318_c0	7.4867	3.61E-38	3.66E-36	Dehydration-responsive element-binding protein 1H OS = Oryza sativa subsp. japonica GN = DREB1H PE = 3 SV = 1
	Comp155879_c0	5.8445	0	0	Dehydration-responsive element-binding protein 1A OS = Oryza sativa subsp. indica GN = DREB1A PE = 3 SV = 1
R3	Comp133037_c0	5.9961	1.46E-05	0.0003279	Dehydration-responsive element-binding protein 1A OS = Oryza sativa subsp. japonica GN = DREB1A PE = 2 SV = 1
	Comp143318_c0	5.5908	4.28E-14	2.24E-12	Dehydration-responsive element-binding protein 1H OS = Oryza sativa subsp. japonica GN = DREB1H PE = 3 SV = 1
	Comp155879_c0	4.8139	2.87E-206	2.62E-203	Dehydration-responsive element-binding protein 1A OS = Oryza sativa subsp. indica GN = DREB1A PE = 3 SV = 1
R4	Comp159413_c1	4.2154	1.47E-06	5.96E-05	Dehydration-responsive element-binding protein 1C OS = Oryza sativa subsp. japonica GN = DREB1C PE = 2 SV = 1
S1	Comp155879_c0	3.1428	2.83E-08	1.24E-06	Dehydration-responsive element-binding protein 1A OS = Oryza sativa subsp. indica GN = DREB1A PE = 3 SV = 1
S3	Comp143318_c0	8.4116	4.08E-05	0.0014065	Dehydration-responsive element-binding protein 1H OS = Oryza sativa subsp. japonica GN = DREB1H PE = 3 SV = 1
	Comp155879_c0	5.2997	1.58E-66	1.62E-63	Dehydration-responsive element-binding protein 1A OS = Oryza sativa subsp. indica GN = DREB1A PE = 3 SV = 1
	Comp159413_c1	3.2936	0.0001694	0.0049795	Dehydration-responsive element-binding protein 1C OS = Oryza sativa subsp. japonica GN = DREB1C PE = 2 SV = 1

(No CBFs were detected in R1, S2 and S4 comparisons)

### KEGG pathway enrichment analysis for DEGs

**Fig. 8:**
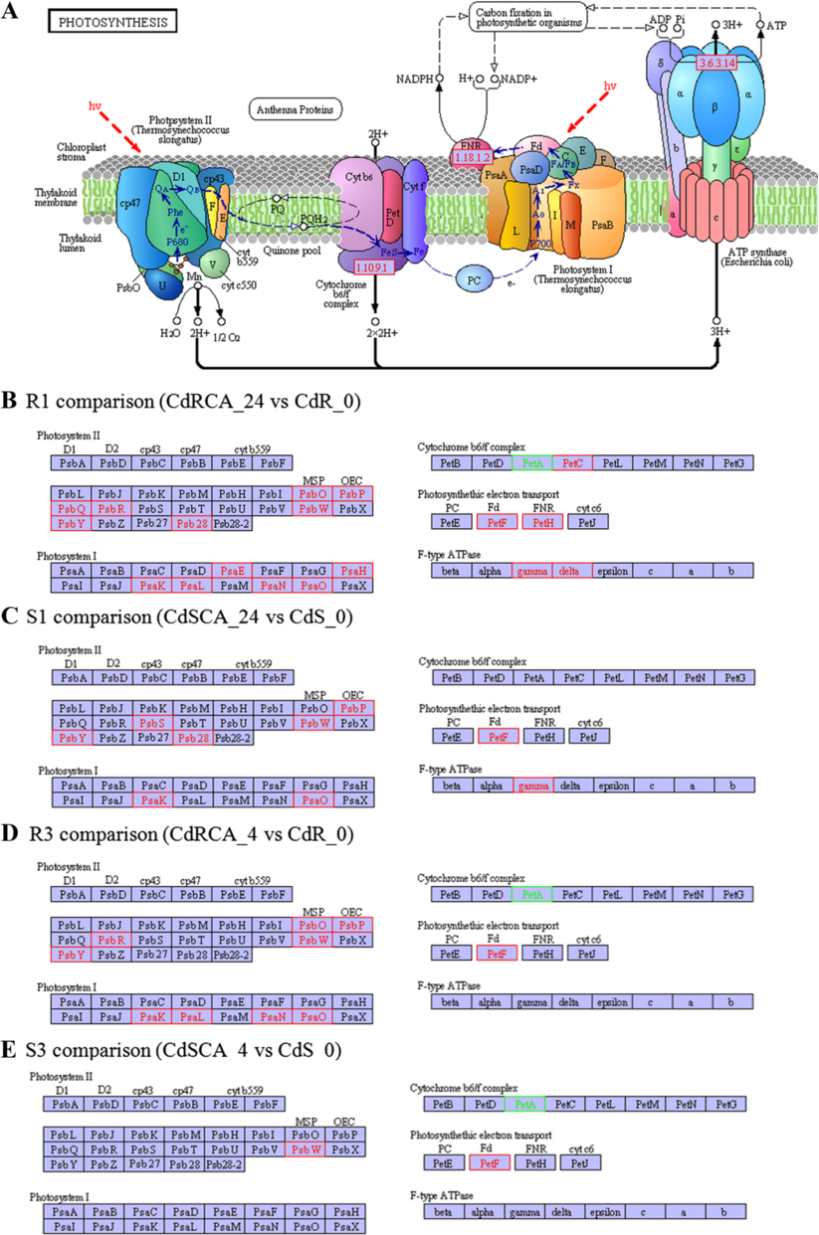
Differentially expressed genes in photosynthesis pathway in different comparisons. Red denotes up-regulated genes and light blue denotes down-regulated genes. **a** The structure of thylakoid; **b**, **c**, **d** and **e** display DEGs in R1 (CdRCA_24 vs CdR_0), S1 (CdSCA_24 vs CdS_0), R3 (CdRCA_4 vs CdR_0), S3 (CdSCA_4 vs CdS_0) comparisons, respectively.

The top-five enriched pathways by DEGs in comparison R1 were photosynthesis, photosynthesis - antenna proteins, nitrogen metabolism, carbon fixation pathways in prokaryotes and carotenoid biosynthesis (Additional file 7). The rich factor for the above five pathways was 49.00, 46.00, 31.43, 28.57, and 30.43 %, respectively, while an equivalent order for the S1 comparison was 16.98, 19.2, 22.86, 10.71 and 17.39, respectively (Additional file 7). The top-five enriched pathways and corresponding rich factor in the comparison R3 were as follows: photosynthesis-antenna proteins (38.46 %), carotenoid biosynthesis (34.78 %), glycerolipid metabolism (20.59 %), galactose metabolism (20.63 %) and photosynthesis (20.75 %). By contrast, the value for the above pathway was 0, 8.70, 2.94, 3.17 and 3.77 %, respectively, in S3 comparison (Additional file 7). As for the photosynthesis pathway, there are 53 genes annotated as involved in this pathway. Of the 53 genes, 26 genes were identified as DEGs in the R1 comparison. Interestingly, among the 26 differentially expressed genes, 25 were induced and only one was down-regulated. In contrast, only 9 genes were found to be up-regulated in the S1 comparison. Likewise, there are 10 up-regulated expressed genes along with one down-regulated gene in the photosynthesis pathway in the R3 comparison, whereas it was found that only two and one were identified as up- and down-regulated genes, respectively (Fig. 8). Moreover, it was unexpectedly discovered that 23 photosynthesis related genes were all down-regulated by freezing temperature without prior CA in R genotype.

### Validate the DEGs by real-time RT-PCR analysis

To validate the expression data obtained from RNA-sequencing, 10 DEGs were selected for confirmation by real-time RT-PCR analysis. The qRT-PCR results showed a strong correlation with the RNA-seq-generated data (Pearson correlation coefficients *r* = 0.878; Additional file 8).

## Discussion

### Global patterns of transcription in response to low temperature

The data available on the molecular basis of the bermudagrass response to low temperature is very limited. In recent years, the development of novel high-throughput sequencing has provided an opportunity to identify cold-related genes in different species by de novo assembly or mapping, thereby contributing to elaborate the molecular mechanism underlying the response to low temperature [8–12]. In the present study, ten bermudagrass cDNA libraries were constructed and sequenced using the Illumina HiSeq™ 2000 platform, and a large number of DEGs were identified. The number of DEGs detected was as follows: R1 3295 (1398 up- and 1897 down-regulated), R2 3391 (1595 up- and 1796 down-regulated), R3 2830 (1194 up- and 1636 down-regulated), R4 1595 (809 up- and 786 down-regulated), R5 4315 (1717 up- and 2598 down-regulated), S1 1793 (983 up- and 810 down-regulated), S2 4799 (2122 up- and 2677 down-regulated), S3 1331 (718 up- and 613 down-regulated), S4 937 (546 up- and 391 down-regulated) and S5 269 (127 up- and 142 down-regulated). Overall, the number of DEGs in the R genotype was larger than that in the S genotype under various cold treatments. The results from parallel comparisons R1 and S1 revealed that more DEGs were detected in comparison R1 (3295) than that in S1 (1793), suggesting that global gene expressions were more quickly initiated in the R genotype than those in S genotype, when they were exposed to cold stress. From the hierarchical clustering analysis, it was found that the S genotype began to appear in a similar model like CdRCA_24 when they were exposed to cold for 48 h (CdSCA_48), further supporting the hypothesis that the S genotype triggered gene expression more slowly than that of the R genotype under cold stress. Besides, there were more DEGs identified in comparisons R3 and S3, which underwent a prior cold acclimation (CA) for 48 h, as compared to the treatments which didn’t undergo CA (R4 and S4), respectively.

Our results further provide additional evidence supporting that plants could obtain enhanced tolerance to freezing temperature when they undergo CA process, and that the acquired resistance may be attributed to a large alteration of global patterns of gene transcription in CA process. However, there was a big difference between R and S genotypes during the CA process as indicated that R5 (CdRCA_4 vs CdRNA_4) has 4315 DEGs (1717 up- and 2598 down-regulated), but only 269 DEGs (127 up- and 142 down-regulated) were identified in S5 (CdSCA_4 vs CdSNA_4) treatment. These results suggest that the R genotype could more effectively activate gene expression during CA process, and thereby better respond to freezing temperature, as compared to S genotype. It was speculated that the prerequisite for plants to obtain enhanced tolerance to freezing through a cold acclimation process is that the plant needs appropriate cold resistance levels.

### Low temperature sensing and signaling genes

It has been shown that various abiotic stresses, including cold, can trigger intracellular changes in free Ca^2+^ concentration, thereby generating the so-called Ca^2+^ signature, which can be sensed by Ca^2+^ sensors and then transduced through the interaction with their target protein to regulate the expression of stress-responsive genes [29–33]. There are Ca^2+^ sensor proteins of three major classes: CDPK, CaM (CML) and CBL [29, 31, 32]. Here, six, one, two and four CMLs were identified as significant DEGs in R1, R2, R3 and R4 comparisons, respectively. Among these 13 CMLs, 9 were found to be down-regulated, indicating that some reduced *CML* genes expression may contribute to enhance plant tolerance to cold stress. Not surprisingly, it was revealed that over-expression of Arabidopsis CaM3 impairs cold induction of RD29A, KIN1 and KIN2 [34]. One *CML* gene (comp147675_c0) was down-regulated in R1, R2, and R3 comparisons. Another *CML* gene (comp135890_c1) transcript was decreased in R1 and R3 comparisons, while it unexpectedly increased in R4 comparison. Likewise, the expression of comp148637_c0 and comp152137_c1 *CML* genes was significantly down-regulated in the R1 comparison, but induced in R4 comparison. These results suggest that CML family proteins may play different roles in the CA process and freezing response with or without a prior CA. Furthermore, no *CML* genes were identified in S3 and S4 comparison in the S genotype, implying that CML family proteins may function as important signaling responders in conferring bermudagrass tolerance to freezing temperature.

The Arabidopsis and rice genomes harbor 34 and 29 CDPK-encoding genes, respectively [35, 36]. CDPKs have been identified as being involved in cold signaling. OsCPK7/OsCDPK13 is activated by cold treatment [37] and overexpression of either OsCPK7/OsCDPK13 or OsCPK13/OsCDPK7 improves cold tolerance in transgenic rice [37, 38]. In the present study, there were many CDPKs identified as DEGs in each comparisons. One *CDPK* gene (comp156791_c0) transcript is accumulated in comparisons R1, R2, R3 and S2, suggesting that the gene may be not only involved in CA process in both R and S genotypes, but also exclusively involved in the freezing response through prior CA in the R genotype in bermudagrass.

The CBL proteins are characterized as a group of plant calcium sensors that could exclusively interact with CIPK proteins. The CBL-CIPK signaling components constitute a specific regulatory network of Ca^2+^ signaling in plant cells [39–41]. Many CBLs and CIPKs have been identified as being involved in plant responses to various abiotic stresses. However, there are few reports on the CBL-CIPK involved in cold stress responses to date. Previous studies have revealed that AtCBL1 is involved in cold response [42, 43]. CIPK3 and CIPK7 were reported to be involved in response to cold stress [39, 42]. Here, two *CBL* genes were identified as DEGs. One *CBL* gene (comp151010_c0) was induced in the following comparisons, R1, R2, R3 and S2. Another *CBL* gene (comp151988_c1) was induced in R4 comparisons, suggesting that the gene may be involved in plant response to chilling stress without a prior CA. There are 46 and 27 DEGs encoding CIPK found in comparisons of cold-resistant and -sensitive genotypes of bermudagrass, respectively. These differentially cold-regulated CBL-CIPK components may be useful for breeding cold-resistant bermudagrass in the future.

### Major classes of TF involved in the response to low temperature

It has been well established that transcription factors (TFs) play important roles in response to different abiotic and biotic stresses. Here, members of various low temperature-responsive transcription factor (TF) families were identified as DTGs in the treatments involved in a process of low temperature acclimation or freezing with or without CA process.

NAC (NAM, ATAF, and CUC) is a plant specific transcription factor family with diverse roles in plant development and in response to abiotic stresses [44–49]. Hu et al. (2008) reported that over-expression of a stress-responsive NAC gene, *SNAC2*, increases rice tolerance to cold and salt [50]. Similarly, overexpression of *TaNAC2* resulted in enhanced tolerances to salt, drought and freezing stresses in Arabidopsis [51]. More recently, Banana MaNAC1 was proven to be a direct target of MaICE1 and involved in cold stress through interacting with MaCBF1 [52]. Here, comp148886_c0 and comp150085_c0 were found to be induced by cold at higher folds in the R genotype than that in the S genotype, suggesting that the two genes may play an important function in conferring R genotype with enhanced cold tolerance, and should be the focus of future studies in bermudagrass.

WRKY TFs are a large family of regulators involved in various developmental and physiological processes, especially in response to diverse biotic and abiotic stresses. Recently, the results from high-throughput transcriptomic analyses have identified that 61 of the *Populus WRKY* genes were induced by various biotic and abiotic treatments, including cold [53]. Transgenic rice over-expressing *OsWRKY76* led to drastically increased susceptibility to *M. oryzae*, but enhanced tolerance to cold stress [54]. In the present study, two *WRKY* genes, comp160681_c0 and comp160771_c0, were induced in the R1, R2, R3 and S2 comparisons, suggesting that these two WRKY proteins play essential roles in the CA process in both R and S genotypes, but specifically involved in the response to the freezing treatment in plants with prior exposure to CA in the R genotype, suggesting its key roles in conferring bermudagrass enhanced freezing tolerance in the R genotype after CA, compared to the S genotype. Moreover, one *WRKY* gene (comp160978_c0) was examined to be significantly up-regulated in the R4 comparison, but not induced in the S4 comparison, indicating that its distinctive function was involved in the R genotype response to chilling without the CA process. These results provide new information that the identified *WRKY* genes from cold-resistant bermudagrass may serve as a target gene for breeding new varieties in future.

To date, plant bHLH TFs have been demonstrated to function as transcription regulators involved in a diversity of biological processes, including flowering [55], trichome development [56], root hair formation and development [57, 58], nodule vascular patterning [59] and the photo-induced signal transduction [60]. Furthermore, bHLH TFs are involved in the plant response to various abiotic stresses, including drought [61], cold [4, 62, 63] and iron deficiency [64]. However, although only a few bHLHs have been identified to be involved in cold tolerance mainly in model plant, cold responsive bHLHs needs further identification, and the underlying mechanisms need further elucidation. In recent years, using RNA-Seq and digital gene expression (DGE) technologies, low temperature induced *bHLH* genes have been identified in grape (*Vitis amurensis* and *Vitis vinifera*) [65], Chrysanthemum (*Chrysanthemum morifolium*) [11] and tea (*Camellia sinensis*) [10]. Here, we also identified cold induced bHLHs in bermudagrass. These genes may play important roles in the enhanced cold hardiness of bermudagrass and should be the focus of future studies in bermudagrass. CBF TFs have been best proven to play primary roles in response to cold stress. Six *CBFs* have been identified in *Arabidopsis*, and three of them, namely, *CBF1/DREB1B*, *CBF2/DREB1C*, and *CBF3/DREB1A*, have been proved to play a primary role in cold acclimation [66–72]. In our present study, *CBFs* specifically and highly expressed in R genotype were identified, and the results contributed to the understanding of the mechanism of bermudagrass response to cold.

### Enriched KEGG pathway participated in response to low temperature

Photosynthesis is regarded as a highly integrated and regulated process which is highly sensitive to environmental changes, because it needs to balance between the light energy absorbed by the photosystems and the energy consumed by metabolic sinks of the plant [73]. It is clear that optimum plant growth and development require a balance in the rates of source versus sink processes. However, cold stress can lead to an imbalance between the source of energy and the metabolic sink, thus requiring photosynthesis adjustments to maintain the balance of energy flow [74]. Previous studies have also reported that low-temperature modulation of the photosynthetic apparatus may be an important factor during the induction of freezing resistance in cereals [75]. As described above, our results revealed that the R genotype may better respond to chilling and freezing, with prior CA, through activating photosynthesis pathway related gene expression, in contrast to the S genotype.

In our present study, it was found that galactose metabolism was identified as enriched pathways in the R3 comparison with the rich factor reached 34.78 %, but just 3.17 % in the S3 comparison, suggesting that galactose metabolism plays essential roles in conferring plant improved tolerance to cold stress. Similarly, a targeted metabolite analysis of two rice genotypes, contrasting in chilling tolerance, revealed that chilling-tolerant genotype accumulated more galactose than that of chilling sensitive genotype during chilling stress [76].

## Conclusions

In conclusion, this study provided the first large-scale transcriptome dataset in bermudagrass in response to low-temperature stress. A total of 326,435 contigs were obtained and 121,166 unigenes were assembled. The differentially expressed genes mainly belonged to low temperature sensing, signaling-related genes, functional proteins and transcription factors, many of which were specifically or predominantly expressed in R genotype under cold treatments. It was also revealed that global gene expressions were initiated more quickly in the R genotype than those in the S genotype, when they were subjected to cold acclimation, and that the R genotype could activate gene expression more effectively to respond to the freezing temperature after CA process than the S genotype. These findings will contribute to understanding the molecular mechanism of bermudagrass response to low temperature.

## Additional files

Additional file 1:
**The phenotypic difference of cold-tolerant (R) and -sensitive(S) bermudagrass genotypes after cold treatment**. (tif 1.55 MB) Additional file 2:
**List of DEGs could be assigned as a GO term in different comparisons.** (rar 1.92 MB) Additional file 3:
**Gene Ontology (GO) classification of the DEGs identified in different comparison between a pair of libraries.** (rar 4.10 MB) Additional file 4:
**The details of the KEGG classification of the different comparisons.** (rar 62.4 KB) Additional file 5:
**The details of DTGs involved in Ca** ^**2+**^ **signalling pathway.** (xls 100 KB) Additional file 6:
**The details of DTGs associated with CIPK.** (doc 114 KB) Additional file 7:
**The enriched pathways by DEGs and rich factor in comparisons.** (xls 15.0 KB) Additional file 8:
**The correlation analysis between RNA-seq-generated data and qRT-PCR confirmed results.** (tif 102 KB)

## Abbreviations

R: Cold-resistant
S: Cold-sensitive
CA: Cold acclimation
DEG: Differential expression gene
CBF: C-repeat (CRT)-binding factor
GO: Gene Ontology
RT-PCR: Reverse transcription-polymerase chain reaction
CBP: Calcium-binding protein
CML: Calmodulin-like protein
CDPK: Calcium-dependent protein kinase
CBL: Calcineurin B-like protein
CIPK: CBL-interacting protein kinases
CBRLK: Calmodulin-binding receptor like kinases
