# Supplementary figures and images for: A transcriptomic analysis of bermudagrass (Cynodon dactylon) provides novel insights into the basis of low temperature tolerance
Source: BMC Plant Biol. 2015 Sep 11;15:216. doi: 10.1186/s12870-015-0598-y (PMC4566850; doi:10.1186/s12870-015-0598-y)

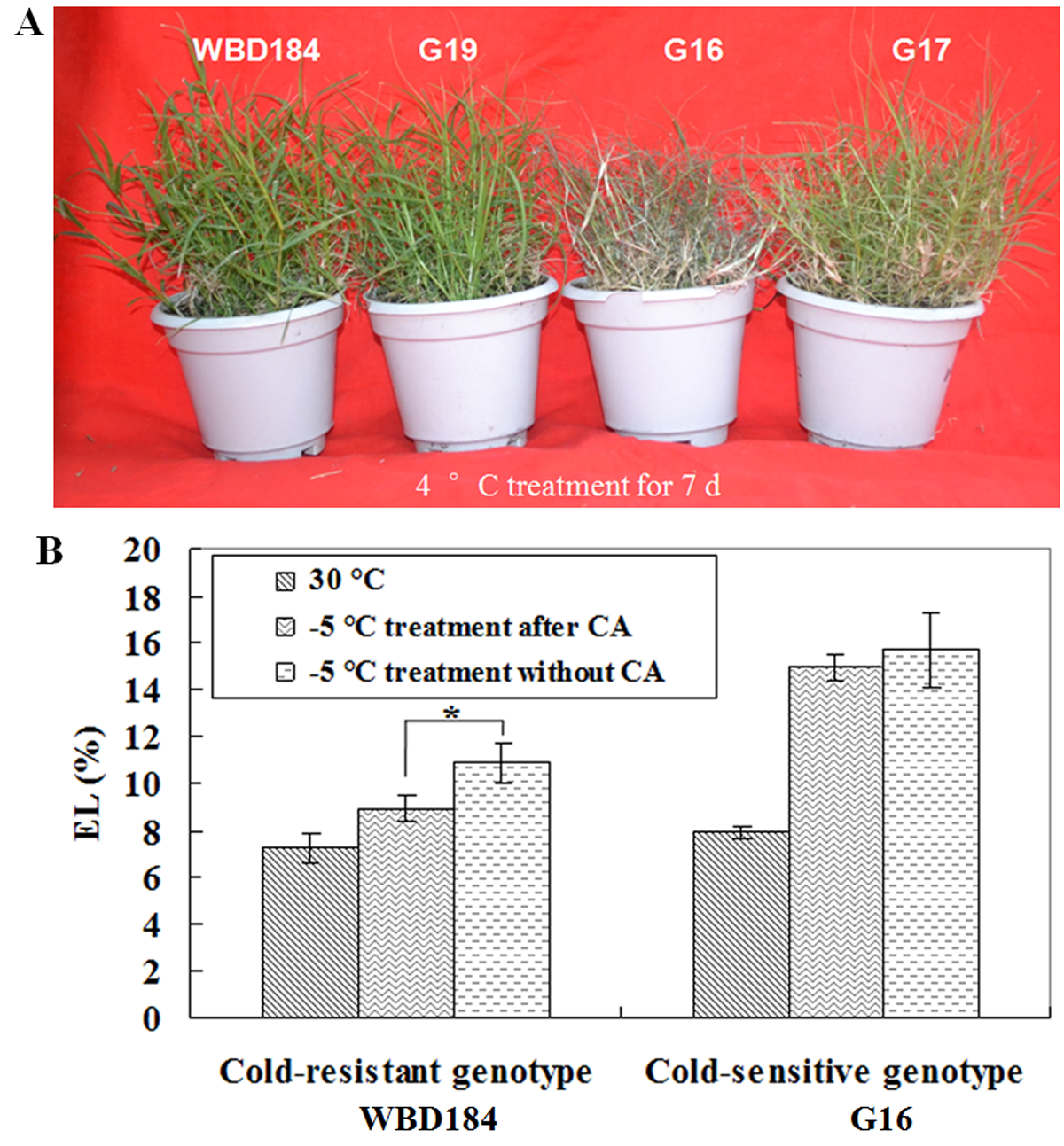

Supplement: Additional file 1: — The phenotypic difference of cold-tolerant (R) and -sensitive(S) bermudagrass genotypes after cold treatment. (tif 1.55 MB) [file 12870_2015_598_MOESM1_ESM.tif]

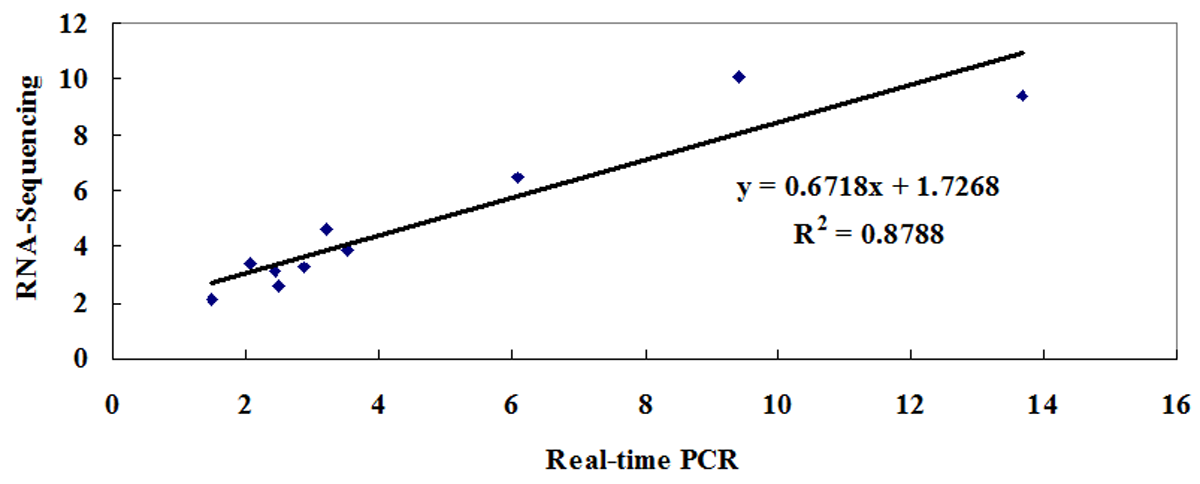

Supplement: Additional file 8: — The correlation analysis between RNA-seq-generated data and qRT-PCR confirmed results. (tif 102 KB) [file 12870_2015_598_MOESM8_ESM.tif]
