# Supplementary material for: A transcriptomic analysis of bermudagrass (Cynodon dactylon) provides novel insights into the basis of low temperature tolerance
Source: BMC Plant Biol. 2015 Sep 11;15:216. doi: 10.1186/s12870-015-0598-y (PMC4566850; doi:10.1186/s12870-015-0598-y)
Supplement: Additional file 6: — The details of DTGs associated with CIPK. (doc 114 KB) [file 12870_2015_598_MOESM6_ESM.doc]

The differential gene expression of *CIPK* genes in each comparison

| Comparison | GeneID | log2  ratio | Up-down regulation | P-value | q-value | Gene description |
| --- | --- | --- | --- | --- | --- | --- |
| R1 | comp159745_c2 | 1.5177 | Up | 0.0001038 | 0.0015526 | CBL-interacting protein kinase 09 [Sorghum bicolor] |
|  | comp151185_c0 | 3.3324 | Up | 6.13E-11 | 1.86E-09 | CBL-interacting protein kinase 5 (Oryza sativa) |
|  | comp151991_c1 | -1.9372 | Down | 1.33E-11 | 4.24E-10 | CBL-interacting protein kinase 12 [Sorghum bicolor] |
|  | comp153204_c0 | -2.5728 | Down | 4.84E-106 | 1.48E-103 | CIPK-like protein 1 [Zea mays] |
|  | comp154876_c0 | -2.7953 | Down | 7.55E-18 | 3.58E-16 | CBL-interacting protein kinase 32 [Sorghum bicolor] |
|  | comp154974_c1 | -1.7562 | Down | 0.0003128 | 0.0042672 | CBL-interacting protein kinase 24 [Sorghum bicolor] |
|  | comp156266_c0 | -6.3007 | Down | 3.03E-104 | 8.82E-102 | CBL-interacting protein kinase 04 [Sorghum bicolor] |
|  | comp156854_c0 | -3.5077 | Down | 2.20E-141 | 9.66E-139 | CBL-interacting protein kinase 29 [Triticum aestivum] |
|  | comp158331_c0 | -1.7382 | Down | 1.38E-107 | 4.33E-105 | CBL-interacting protein kinase 25 [Sorghum bicolor] |
|  | comp159481_c1 | -4.088 | Down | 9.95E-125 | 3.74E-122 | CBL-interacting protein kinase 14 [Triticum aestivum] |
|  | comp160040_c0 | -1.3898 | Down | 4.85E-13 | 1.72E-11 | putative CBL-interacting protein kinase family protein [Zea mays] |
|  | comp160675_c1 | -1.8798 | Down | 1.33E-09 | 3.69E-08 | CBL-interacting protein kinase 31 [Sorghum bicolor] |
| R2 | comp151708_c0 | 3.5945 | Up | 3.23E-31 | 2.64E-29 | TPA: putative CBL-interacting protein kinase family protein isoform 1 [Zea mays] |
|  | comp153204_c0 | -2.5795 | Down | ######## | ######## | CIPK-like protein 1 [Zea mays] |
|  | comp154876_c0 | -2.643 | Down | 1.42E-16 | 6.56E-15 | CBL-interacting protein kinase 32 [Sorghum bicolor] |
|  | comp154974_c1 | -1.8585 | Down | 0.000262 | 0.003564 | CBL-interacting protein kinase 24 [Sorghum bicolor] |
|  | comp156266_c0 | -3.5131 | Down | 1.55E-86 | 4.19E-84 | CBL-interacting protein kinase 04 [Sorghum bicolor] |
|  | comp156854_c0 | -2.3868 | Down | 7.81E-97 | 2.51E-94 | CBL-interacting protein kinase 29 [Triticum aestivum] |
|  | comp157967_c0 | -1.1089 | Down | 0.000211 | 0.002923 | PA: putative CBL-interacting protein kinase family protein [Zea mays] |
|  | comp159481_c1 | -3.03 | Down | 4.73E-99 | 1.56E-96 | CBL-interacting protein kinase 14 [Triticum aestivum] |
|  | comp160675_c1 | -1.0751 | Down | 7.47E-05 | 0.001132 | CBL-interacting protein kinase 31 [Sorghum bicolor] |
|  | comp151708_c0 | 3.5945 | Up | 3.23E-31 | 2.64E-29 | putative CBL-interacting protein kinase family protein isoform 1 [Zea mays] |
|  | comp153204_c0 | -2.5795 | Down | 7841E-103 | 2774E-101 | CIPK-like protein 1 [Zea mays] |
|  | comp154876_c0 | -2.643 | Down | 1.42E-16 | 6.56E-15 | CBL-interacting protein kinase 32 [Sorghum bicolor] |
|  | comp154974_c1 | -1.8585 | Down | 0.0002624 | 0.0035639 | CBL-interacting protein kinase 24 [Sorghum bicolor] |
|  | comp156266_c0 | -3.5131 | Down | 1.55E-86 | 4.19E-84 | CBL-interacting protein kinase 04 [Sorghum bicolor] |
|  | comp156854_c0 | -2.3868 | Down | 7.81E-97 | 2.51E-94 | CBL-interacting protein kinase 29 [Triticum aestivum] |
|  | comp157967_c0 | -1.1089 | Down | 0.0002112 | 0.002923 | TPA: putative CBL-interacting protein kinase family protein [Zea mays] |
|  | comp159481_c1 | -3.03 | Down | 4.73E-99 | 1.56E-96 | CBL-interacting protein kinase 14 [Triticum aestivum] |
|  | comp160675_c1 | -1.0751 | Down | 7.47E-05 | 0.0011322 | CBL-interacting protein kinase 31 [Sorghum bicolor] |
|  | comp152321_c0 | 1.2427 | Up | 4.87E-09 | 1.30E-07 | CIPK-like protein 1, putative, expressed [Oryza sativa Japonica Group] |
| R3 | comp151708_c0 | 1.6019 | Up | 1.32E-05 | 0.000298 | TPA: putative CBL-interacting protein kinase family protein isoform 1 [Zea mays] |
|  | comp151991_c1 | -1.4913 | Down | 1.11E-06 | 2.93E-05 | CBL-interacting protein kinase 12 [Sorghum bicolor] |
|  | comp153204_c0 | -2.595 | Down | 8.02E-94 | 2.94E-91 | CIPK-like protein 1 [Zea mays] |
|  | comp154876_c0 | -2.5066 | Down | 3.24E-14 | 1.71E-12 | CBL-interacting protein kinase 32 [Sorghum bicolor] |
|  | comp156266_c0 | -5.4412 | Down | 1.138E-104 | 4.6862E-102 | CBL-interacting protein kinase 04 [Sorghum bicolor] |
|  | comp156854_c0 | -3.2304 | Down | 3.97E-122 | 2.05E-119 | CBL-interacting protein kinase 29 [Triticum aestivum] |
|  | comp158331_c0 | -1.0992 | Down | 1.04E-36 | 1.33E-34 | CBL-interacting protein kinase 25 [Sorghum bicolor] |
|  | comp159481_c1 | -3.3634 | Down | 6.18E-102 | 2.43E-99 | CBL-interacting protein kinase 14 [Triticum aestivum] |
|  | comp160040_c0 | -1.2624 | Down | 3.14E-08 | 1.01E-06 | TPA: putative CBL-interacting protein kinase family protein [Zea mays] |
|  | comp160675_c1 | -1.7916 | Down | 1.96E-07 | 5.70E-06 | CBL-interacting protein kinase 31 [Sorghum bicolor] |
|  | comp152321_c0 | 1.1788 | Up | 3.99E-12 | 1.83E-10 | CIPK-like protein 1, putative, expressed [Oryza sativa Japonica Group] |
| R4 | comp151708_c0 | 2.0463 | Up | 2.33E-09 | 1.39E-07 | TPA: putative CBL-interacting protein kinase family protein isoform 1 [Zea mays] |
|  | comp153204_c0 | -2.392 | Down | 3.16E-83 | 2.61E-80 | CIPK-like protein 1 [Zea mays] |
|  | comp156266_c0 | -1.9227 | Down | 1.38E-38 | 4.00E-36 | CBL-interacting protein kinase 04 [Sorghum bicolor] |
|  | comp159481_c1 | -1.7672 | Down | 1.87E-43 | 6.01E-41 | CBL-interacting protein kinase 14 [Triticum aestivum] |
| S1 | comp153204_c0 | 2.0566 | Up | 1.20E-94 | 1.33E-91 | CIPK-like protein 1 [Zea mays] |
|  | comp156266_c0 | 1.585 | Up | 2.27E-32 | 4.75E-30 | CBL-interacting protein kinase 04 [Sorghum bicolor] |
|  | comp158125_c0 | 1.4941 | Up | 2.77E-49 | 9.78E-47 | CBL-interacting protein kinase 21 [Sorghum bicolor] |
|  | comp158331_c0 | 1.5868 | Up | 9.0087E-152 | 2.1256E-148 | CBL-interacting protein kinase 25 [Sorghum bicolor] |
|  | comp159461_c1 | 1.1095 | Up | 4.70E-06 | 0.0001405 | CBL-interacting serine/threonine-protein kinase 15 [Zea mays] |
|  | comp159481_c1 | 2.0169 | Up | 5.43E-78 | 3.81E-75 | CBL-interacting protein kinase 14 [Triticum aestivum] |
|  | comp159745_c1 | 1.5154 | Up | 1.47E-10 | 8.39E-09 | TPA: putative CBL-interacting protein kinase family protein [Zea mays] |
| S2 | comp151185_c0 | 2.5179 | Up | 4.70E-06 | 5.91E-05 | CBL-interacting protein kinase 5 [Oryza sativa Japonica Group] |
|  | comp151991_c1 | -1.3306 | Down | 1.46E-08 | 2.44E-07 | CBL-interacting protein kinase 12 [Sorghum bicolor] |
|  | comp152118_c1 | 2.1428 | Up | 3.11E-05 | 0.000344 | CBL-interacting protein kinase 28 [Sorghum bicolor] |
|  | comp153204_c0 | -1.8605 | Down | 5.81E-40 | 3.96E-38 | CIPK-like protein 1 [Zea mays] |
|  | comp154876_c0 | -3.3253 | Down | 1.04E-21 | 3.92E-20 | CBL-interacting protein kinase 32 [Sorghum bicolor] |
|  | comp154974_c1 | -2.5927 | Down | 1.22E-09 | 2.25E-08 | CBL-interacting protein kinase 24 [Sorghum bicolor] |
|  | comp156266_c0 | -5.1847 | Down | 2.07E-57 | 2.11E-55 | CBL-interacting protein kinase 04 [Sorghum bicolor] |
|  | comp156854_c0 | -3.2325 | Down | 3.2609E-123 | 7.7927E-121 | CBL-interacting protein kinase 29 [Triticum aestivum] |
|  | comp158331_c0 | -1.5321 | Down | 2.38E-112 | 4.92E-110 | CBL-interacting protein kinase 25 [Sorghum bicolor] |
|  | comp159481_c1 | -3.155 | Down | 2.27E-55 | 2.18E-53 | CBL-interacting protein kinase 14 [Triticum aestivum] |
|  | comp159745_c1 | 1.0781 | Up | 0.0002495 | 0.0023489 | TPA: putative CBL-interacting protein kinase family protein [Zea mays] |
|  | comp160040_c0 | -1.793 | Down | 8.73E-33 | 4.91E-31 | TPA: putative CBL-interacting protein kinase family protein [Zea mays] |
|  | comp152321_c0 | 1.7028 | Up | 4.77E-08 | 7.56E-07 | CIPK-like protein 1, putative, expressed [Oryza sativa Japonica Group] |
| S3 | comp151708_c0 | 1.9887 | Up | 1.27E-13 | 1.46E-11 | TPA: putative CBL-interacting protein kinase family protein isoform 1 [Zea mays] |
|  | comp154876_c0 | -1.9199 | Down | 3.88E-10 | 3.18E-08 | CBL-interacting protein kinase 32 [Sorghum bicolor] |
|  | comp159481_c1 | -2.2822 | Down | 1.63E-32 | 5.75E-30 | CBL-interacting protein kinase 14 [Triticum aestivum] |
|  | comp152321_c0 | 1.1971 | Up | 1.53E-08 | 1.01E-06 | CIPK-like protein 1, putative, expressed [Oryza sativa Japonica Group] |
| S4 | comp154876_c0 | -1.4487 | Down | 2.55E-08 | 2.03E-06 | CBL-interacting protein kinase 32 [Sorghum bicolor] |
|  | comp158331_c0 | 1.2959 | Up | 2.915E-156 | 3.3054E-152 | CBL-interacting protein kinase 25 [Sorghum bicolor] |
|  | comp152321_c0 | 1.4121 | Up | 1.83E-08 | 1.48E-06 | CIPK-like protein 1, putative, expressed [Oryza sativa Japonica Group] |
